# Global assessment of childhood growth monitoring: cross-sectional survey of national policies and practices

**DOI:** 10.7189/jogh.16.04034

**Published:** 2026-02-06

**Authors:** Annariina Koivu, Ulla Ashorn, Elaine Borghi, Andreas Hasman, Purnima Menon, Aman Pulungan, Julie Ruel-Bergeron, Linda Shaker-Berbari, Madhumita Singh, Naveen Thacker, Wilson Milton Were, Kaisa Ylikruuvi, Per Ashorn, International Growth Monitoring Survey Consortium, Osama Abdi, Madina Ali Abdirahman, Belen Aguirrezabalaga, Basim Al-Zoubi, Mona Alameh, Cecilia D. Alinea, Christine Jane B. Almira, Hind Alsharhan, Abdulmajeed AlSubaihin, Rola Alzir, Beatrice Amadi, Michael Anastasiades, Kim Ang, Ananias Antonio, Sofijanova Aspazija, Simon Jonas Ategbo, Svitlana Austin, Damte Shimelis Awoke, Khamisa Ayoub, Ingrid Pamela Báez Echeverría, Shamsov Bakhtovar, Cecilia Barragán, Nigar Bayramova, Ramush Bejiqi, Nellie VT Bell, Bruno Bindamba Senge, Nardos Birru, Asma Bouaziz, María Catalina Carvajal, Alvin SM Chang, Jean-Pierre Chanoine, Olga Cirstea, Natália Maria Ferreira da Conceiçâo Rodrigues, Laura María Cristales Telón, Lovely Daisy, Ibrahima Sory Diallo, Ana Lucía Díez Recinos, Željka Draušnik, Ekanem Ekure, Mercedes Esquivel Lauzurique, Ali Faraj Ali Nassr, Julia Fernández Monge, Kouéta Fla, Amorissani Folquet, Christophe Gnimi, Jean Chrysostome Gody, Madonna Grimes, Nadia Guellouz, Sahar Idelbi, Violeta Iotova, Majed Abu Jaish, Tuomas Jartti, Mari-Louie Jeffery, Pawana Kayastha, Mediatrice Kiburente, Georgios Konstantinidis, Anne Mei-Kwun Kwok, Maja Lang Morović, Agnès Linglart, Phim Loan, Kvashnina Lyudmila, Fiawoo Mawouto, Emmie W Mbale, Hubert Désiré Mbassi Awa, María José Mendoza, Gladys Anabella Miranda Fuentes, Ladda Mo-Suwan, Annang Giri Moelyo, Claudia Montesinos Ramirez, Paul Moscoso, Bouraima Mouawiyatou, Yeva Movsesyan, Florence Mtawale, Aida Mujkić, Mehreen Mujtaba, Aimée Mupuala, Pius David Muzzazzi, Barbara Nalubanga, Leyla Namazova-Baranova, Gorban Nataliya, Rute Neves, Pie Nibirantije, Akhmedova Nilufar, Fidele Nkezabahizi, Dler Abdulkhaleq Nooruldeen, Cecelia J. Nuta, Azubuike Benjamin Nwako, Emmanuel Oppong, Fartun Abdullahi H Orey, Altagracia Páez, Huynh Nam Phuong, Mariana del Pino, Doina-Anca Plesca, Jorge Rada Noriega, Tahiana Razafindrakoto, Macarena Riquelme Rivera, María Inés Romero, Marysol Ruilova, Elieth Rumanyika, Masood Sadiq, Haroon Saloojee, Setshedi Sebata, Amela Selimovic, Virendra RS Singh, Selva Kumar Sivapunniam, Santosh Soans, Dirceu Sole, Félix Sonon, Chan Sophal, Adriana Sosa Botana, Shayirbek Alibaevich Sulaimanov, Mariam Sylla, Lila Bikram Thapa, Fathimath Thohira, Vaidotas Urbonas, Ruth Aburto Vallejos, Laura Elizabeth Vásquez Muñoz, Verónica Véliz Rojas, Sergio Venturino, V Pujitha Wickramasinghe, Wei Xiang, Hamda Omar Yousuf, Antipkin Yuriy, Fatima Mohammed Yusuf, Leela Keculah Zaizay, Yue Zhang, Magaly Zurita Villazón, Siniketiwe Zwane

**Affiliations:** 1 Centre for Child, Adolescent and Maternal Health Research, Tampere University, Tampere, Finland; 2 Department of Nutrition and Food Safety, World Health Organization, Geneva, Switzerland; 3 Child Nutrition and Development, United Nations Children’s Fund, New York, New York, USA; 4 Poverty, Health and Nutrition Division, International Food Policy Research Institute, South Asia Office, New Delhi, India; 5 International Paediatric Association, Marengo, Illinois, USA; 6 Department of Child Health, Faculty of Medicine, University of Indonesia, Jakarta, Indonesia; 7 Human Development Network’s Health, Nutrition, and Population, World Bank, Washington D.C., USA; 8 Department of Maternal, Newborn, Child and Adolescent Health and Ageing, World Health Organization, Geneva, Switzerland

## Abstract

**Background:**

Monitoring children’s growth is crucial in paediatric care for early identification of health issues, with the World Health Organization (WHO) advocating for its practice throughout childhood. However, the focus and implementation of growth monitoring vary globally, reflecting different health priorities and practices.

**Methods:**

We conducted a global, cross-sectional, questionnaire-based survey, targeted at representatives of the ministry responsible for growth monitoring and promotion, and at representatives of national paediatric societies.

**Results:**

We obtained responses from 122 countries. Of these, 88% had national growth monitoring guidance, most often issued by the ministry of health. Weight was the most consistently measured early childhood growth monitoring indicator, recorded routinely in 98% of countries during growth monitoring visits for children aged <1 year. The WHO Child Growth Standards were used in 86% of countries. The most common follow-up action for growth faltering was provision of nutritional or health advice, cited by 91% of respondents for children aged <1 year, with advice frequency decreasing as child age increased.

**Conclusions:**

Childhood growth monitoring is widely adopted, but implemented with considerable variation across countries. Strengthening its impact will require standardising indicators, integrating evidence-based guidelines into primary care, and ensuring equitable, actionable use across age groups.

Monitoring children’s growth is a standard practice in paediatric care globally. It is based on the well-established understanding that excessively slow or rapid rates of weight or height gain, which are linked to multiple adverse outcomes, can serve as sensitive indicators of a child’s health. By systematically tracking anthropometric measurements such as weight, height, and body mass index (BMI) over time, health service providers can identify deviations from expected growth patterns and intervene early to prevent or manage potential health concerns.

The World Health Organization (WHO) and the United Nations Children’s Fund (UNICEF) recommend growth monitoring throughout childhood and adolescence as a component of broader well-child visits, with the WHO Child Growth Standards used as a reference throughout the process [1,2]. While its importance is widely recognised, the purpose and implementation of growth monitoring vary across countries and remain a subject of debate [3−5]. Some programmes utilise it primarily as an early warning system to identify children at risk of growth faltering (*e.g.* Ethiopia) [6], while others shift the focus to overweight children (*e.g.* Qatar) [7]. The different approaches to the end goals of growth monitoring are reflected in what is measured and which anthropometric indices are calculated. For example, while health workers in Ghana focus on growth trends by tracking weight-for-age over time, those in Nepal tend to rely on a single measurement point to assess underweight, emphasising a child’s current status over their growth trajectory [8].

The components of growth monitoring, such as the use of length measurement, are also being reconsidered in the literature [9−11]. A further ongoing debate relates to the use of a global standard for growth assessment, as opposed to local references [12,13]. Finally, variations in operational factors, including utilisation levels, the accuracy of measurement and interpretation, health system capacity for referrals, and community support, can differ considerably across contexts, and can significantly influence the quality and outcomes of growth monitoring [14−16]. Given this heterogeneity, it is currently challenging not only to assess the impact of growth monitoring but also to clearly define what it currently entails, what it should aim to achieve, and the rationale for its continued use [14].

Understanding how countries implement growth monitoring programmes is a necessary first step in determining how to optimise them and enhance their impact. Because no comprehensive global review of practices and policies has been conducted in more than two decades, we aimed to assess the availability and scope of national guidance on growth monitoring practices, investigate the currently recommended schedules and content of growth assessments, and evaluate the methods used to interpret anthropometric data. Analysing these components is essential for identifying gaps in practice and aligning monitoring strategies with evidence-based recommendations to promote optimal child health outcomes. We further wanted to explore the recommended follow-up actions when growth faltering is detected. While many guidelines emphasise early detection, the actions taken following abnormal growth patterns can vary widely and influence programme outcomes. Overall, our findings would contribute to the publicly available country reports by enabling cross-regional comparisons and the identification of trends and contextual differences, providing independent, empirical evidence to inform policy and practice. Ultimately, they would add to the global discussions on the future directions of childhood growth monitoring that ensure timely and appropriate interventions for children at risk.

## METHODS

### Study design

We conducted a cross-sectional, questionnaire-based survey between October 2024 and June 2025 (Appendix S1 in the **Online Supplementary Document**). The questionnaire was initially drafted by one author (PA) and iteratively revised by all authors. Then, it was piloted with six respondents identified by the UNICEF and the International Paediatric Association (IPA), revised based on their feedback, and collaboratively approved by all authors. Native speakers translated the original English version into French, Spanish, and Arabic, and each translation was subsequently reviewed for accuracy by another native speaker. We re-piloted the online questionnaire on a small sample of participants to assess its clarity and validity. We used Research Electronic Data Capture (REDCap), versions 14 and 15 (Vanderbilt University, Nashville, Tennessee, USA) [17] to transpose the questionnaire to an anonymous survey format for online data collection, and to subsequently store the data at Tampere University. Ethics Committee of the Tampere Region reviewed and approved the study protocol (statement 82/2924).

### Data collection

The questionnaire was targeted at representatives of the ministry responsible for growth monitoring and promotion in 195 countries and regions in which the UNICEF operates, as well as representatives of 153 national paediatric societies that are members of the IPA. We aimed to select two possible respondents from most countries to account for variations in national responsibility for growth monitoring and the associated guidelines. In some countries, this responsibility primarily falls on a national ministry, while in others, it rests more with the paediatric community.

The UNICEF sent an invitation and information letter, together with a link to the survey, via regional UNICEF offices to the contact person in the relevant ministry for each target country or area in December 2024. The IPA sent the same information and invitation to the president and secretary of each national paediatric society in October 2024. Both sending organisations selected the language in the contact letter based on the recipient country. Recipients could not change the language of the initial contact letter but could select their preferred language for the participant information page presented at the beginning of the electronic survey. Both at the ministry and at the national paediatric association, the recipient of the initial letter was allowed to respond to it himself or to forward the survey information and link to the expert who would be better positioned to answer the survey questions. There were a minimum of three rounds of follow-up messages after the UNICEF invitation, and five following the IPA invitation, targeting non-respondents or those who had started but not completed the survey. The survey was closed on 6 June 2025.

We asked respondents to complete an online questionnaire comprising 30 items, with the option to select their preferred language. The questionnaire comprised single-response questions (for which response proportions sum to 100%), multiple-response questions (where proportions may exceed 100% due to respondents selecting more than one option), and open-ended questions.

We collected information on the national guidance that provides instructions, the authority implementing the childhood growth monitoring, and the purposes, setting, and contents of growth monitoring. Furthermore, we collected information related to the frequency of growth monitoring conducted during well-child visits, anthropometric measurements taken, anthropometric indices calculated, growth reference used, result interpretation, and follow-up actions. Finally, through open-ended questions, we invited respondents to provide any inputs, comments, or recommendations on growth monitoring in their own words, which we then translated into English for analysis.

### Statistical analysis

The data required minimal cleaning. We analysed the data with *R*, version 4.4.1 (R Core Team, Vienna, Austria) to generate descriptive statistics.

To determine the institutional affiliation of each response, we asked respondents whether they represented a national ministry or another national institution with oversight of growth monitoring, a national paediatric society, or another type of organisation (specified by the respondent). We reviewed and, where appropriate, reclassified responses categorised as ‘other’.

For analysis, we combined the results that came through UNICEF and IPA. If a country provided only one response, we included it in the data set for analysis. In cases where responses were received from both sources, we used the response from the ministry (via UNICEF invitation). This is because the ministries of health are the primary institutions responsible for establishing and enforcing national growth monitoring policies and guidelines. For quality assurance, we calculated the proportion of identical responses between UNICEF and IPA sources in the 29 countries where we received responses from both. The similarity percentage ranged between 57–78%. In case of discrepancies, we treated the differences as minor (Appendix S2 in the **Online Supplementary Document**). Based on this result, we considered it appropriate to merge data from the two different sources.

We disaggregated the data by the United Nations’ world regions [18] to facilitate comparison across geographical contexts. Due to the small number of responses (n = 3), we grouped the response from Oceania with those from Asia. This approach maintained respondent privacy and had a negligible impact on the overall findings.

### Thematic analysis

We analysed open-ended responses using the six-step reflexive inductive thematic analysis proposed by Braun and Clarke [19,20]. One researcher (AK) coded the responses manually in Microsoft Excel for Microsoft 365 (Microsoft Corporation, Redmond, Washington, USA) and subsequently identified emerging themes. Another researcher (UA) then reviewed the codes and themes to ensure validation and reduce bias.

Given the extensiveness of the survey data, we present only a subset of results in this article, with further findings to be published separately.

## RESULTS

**Figure 1:**
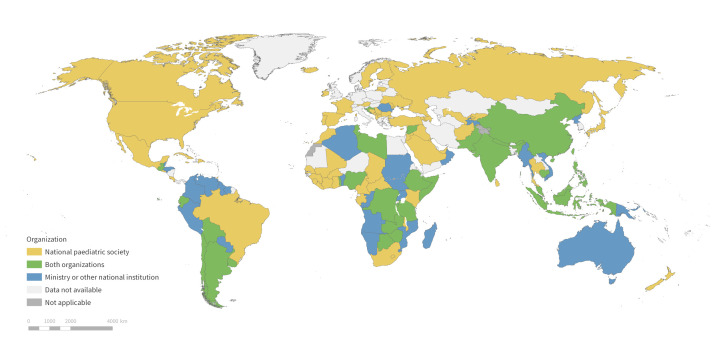
Countries from which at least one completed response to the National Growth Monitoring Survey was received, colour-coded by the type of responding organisation.

We received responses from 122 countries (**Figure 1**), with most coming from Africa (33%), followed by Asia (28%), the Americas (20%), Europe (17%) and Oceania (2%). The regional response rate was the highest for Africa, with at least one response was received from 40 of 54 countries (74%), followed by Asia (68%), the Americas (67%), Europe (48%), and Oceania (21%). In total, 57 countries contacted via UNCIEF and 94 contacted via IPA had a completed response. In 29 countries, responses were received from both sources.

Of all respondents, 47% represented a national ministry, another national institution with oversight on growth monitoring, or UNICEF, while 53% represented a national paediatric society. Most respondents (88%) reported having national growth monitoring guidance in their country, usually issued by a single organisation (74%), but sometimes by two or more organisations/authorities. In 12 countries (10%), respondents reported having no national guidance on growth monitoring.

Almost all (95%) respondents stated that the national guidance was issued by the national ministry responsible for child health, public health, or nutrition. Additionally, 14% of the respondents mentioned guidance by a national paediatric society or association, and 9% mentioned another source. Only one respondent deemed the question not applicable, as growth monitoring was not practised in her/his country.

### Anthropometric measurements

Among anthropometric measurements, weight was reported to be measured always or almost always across all world regions during the first five years of life. Thereafter, the recommended measurement frequency tended to decrease in most areas. Length or height was reported to be regularly measured during the first five years of life in almost all responding countries in the Americas and Europe (92–100%), but less consistently in Asia (84–89%) and in only approximately half of the countries in Africa (48–60%). Similarly to weight, the frequency of length/height measurements tended to decline with increasing age beyond five years in all regions. Head circumference was reported less frequently and was largely limited to the first two years of life, while mid-upper arm circumference (MUAC) measurement appeared to be the least commonly reported (**Table 1**).

**Table 1:** Proportion of measurements reportedly taken always or almost always at growth monitoring visit, by child age and geographic region, n (%)

Variables and age groups	World (n = 122)	Africa (n = 40)	Americas (n = 24)	Asia and Oceania (n = 37)	Europe (n = 21)
Weight					
*0–11 months*	119 (98)	37 (93)	24 (100)	37 (100)	21 (100)
*12–23 months*	116 (95)	36 (90)	23 (96)	36 (97)	21 (100)
*2–4 years*	110 (90)	32 (80)	23 (96)	35 (95)	20 (95)
*5–9 years*	81 (66)	19 (48)	20 (83)	23 (62)	19 (90)
*10–17 years*	75 (61)	17 (43)	19 (79)	21 (57)	18 (86)
Length/height					
*0–11 months*	100 (82)	24 (60)	22 (92)	33 (89)	21 (100)
*12–23 months*	95 (78)	22 (55)	22 (92)	31 (84)	20 (95)
*2–4 years*	92 (75)	19 (48)	22 (92)	31 (84)	20 (95)
*5–9 years*	70 (57)	11 (28)	19 (79)	21 (57)	19 (90)
*10–17 years*	62 (51)	10 (25)	17 (71)	17 (46)	18 (86)
Head circumference					
*0–11 months*	75 (61)	15 (38)	18 (75)	23 (62)	19 (90)
*12–23 months*	55 (45)	10 (25)	13 (54)	19 (51)	13 (62)
*2–4 years*	27 (22)	5 (13)	5 (21)	11 (30)	5 (24)
*5–9 years*	10 (8)	0 (0)	1 (4)	4 (11)	5 (24)
*10–17 years*	6 (5)	0 (0)	1 (4)	2 (5)	3 (14)
MUAC					
*0–11 months*	36 (30)	22 (55)	1 (4)	8 (22)	5 (24)
*12–23 months*	38 (31)	23 (58)	2 (8)	8 (22)	5 (24)
*2–4 years*	28 (23)	15 (38)	1 (4)	7 (19)	5 (24)
*5–9 years*	9 (7)	3 (8)	1 (4)	0 (0)	5 (24)
*10–17 years*	7 (6)	2 (5)	1 (4)	0 (0)	4 (19)

MUAC – mid-upper arm circumference

### Growth reference choice

In the countries where growth monitoring was practised, the most used growth reference for comparison and calculating anthropometric indices for children aged <5 years was the WHO Child Growth Standards, selected by 86% of respondents. Further, 30 countries (25%) reported using a national growth reference or standard, but only 11 of them used it as their sole reference. Usage of a national reference varied significantly by region, being more common in Europe and Asia, while remaining notably low in the Americas and in Africa. Other references, reportedly used by 8% of the countries, included, for example, the Centres for Disease Control and Prevention growth charts, which was used by one country only. The results for children aged >5 years followed a similar pattern, with the exception that the percentage of countries reporting that growth monitoring is not practised in this age group increased to 11% (**Table 2**).

**Table 2:** Growth reference used for comparison and calculating anthropometric indices for children aged <5 y and >5 y by geographic region, n (%)

Growth reference and children’s age in years	World (n = 122)	Africa (n = 40)	Americas (n = 24)	Asia and Oceania (n = 37)	Europe (n = 21)
WHO Child Growth Standards					
*<5*	105 (86)	37 (92)	23 (96)	32 (86)	13 (62)
*>5*	87 (71)	29 (72)	22 (92)	23 (62)	13 (62)
National growth reference/standard					
*<5*	30 (25)	3 (8)	5 (21)	14 (38)	8 (38)
*>5*	28 (23)	1 (2)	5 (21)	13 (35)	8 (38)
Something else					
*<5*	10 (8)	2 (5)	2 (8)	3 (8)	3 (14)
*>5*	10 (8)	3 (8)	1 (4)	4 (11)	2 (10)
Don’t know					
*<5*	0 (0)	0 (0)	0 (0)	0 (0)	0 (0)
*>5*	1 (1)	0 (0)	0 (0)	1 (3)	0 (0)
Not applicable*					
*<5*	1 (1)	1 (2)	0 (0)	0 (0)	0 (0)
*>5*	14 (11)	9 (22)	0 (0)	5 (14)	0 (0)

WHO – World Health Organization

*Growth monitoring is not practised in this age group in the respondent’s country.

### Criteria for growth problems

The most common criterion for identifying problems with the child’s growth was a too low or too high value in the latest weight-for-age Z-score (WAZ), selected by approximately three-quarters of respondents. This was followed by a too low or too high value in the most recent weight-for-length/height Z-score (WLZ/WHZ) (66%) and length/height-for-age Z-score (LAZ/HAZ) (53%). Fewer than half of the respondents cited MUAC values, and changes in growth velocity were selected less frequently. There was, however, regional variation in Africa, where attained WAZ, WLZ/WHZ, and MUAC were most frequently used (each reported by at least 70% of respondents), while in other regions, attained WAZ and LAZ/HAZ were commonly recommended, but MUAC was infrequently (**Table 3**). Six countries indicated that they used only too low or too high values in the latest WAZ to determine whether there is a problem with the child’s growth.

**Table 3:** Criteria used to determine if there is a problem with the child’s growth by geographic region, n (%)

Criteria	World (n = 122)	Africa (n = 40)	Americas (n = 24)	Asia and Oceania (n = 37)	Europe (n = 21)
Too low/high WAZ*	91 (75)	29 (72)	21 (88)	27 (73)	14 (67)
Too low/high WLZ/WHZ	80 (66)	29 (72)	16 (67)	23 (62)	12 (57)
Too low/high MUAC	45 (37)	28 (70)	4 (17)	9 (24)	4 (19)
Too low/high LAZ/HAZ	65 (53)	21 (52)	12 (50)	22 (59)	10 (48)
Too low/high parental-height adjusted LAZ/HAZ	15 (12)	3 (8)	2 (8)	7 (19)	3 (14)
Too low/high change in WHZ	40 (33)	10 (25)	6 (25)	17 (46)	7 (33)
Too low/high change in LAZ/HAZ	40 (33)	7 (18)	9 (38)	16 (43)	8 (38)
Something else	12 (10)	2 (5)	3 (12)	5 (14)	2 (10)
Don’t know	4 (3)	0 (0)	0 (0)	2 (5)	2 (10)
Not applicable†	3 (2)	2 (5)	0 (0)	0 (0)	1 (5)

HAZ – height for age Z-score, LAZ – length for age Z-score, MUAC – mid-upper arm circumference, WAZ – weight for age Z-score, WHZ – weight for height Z-score, WLZ – weight for length Z-score

*Value that is below or above a nationally or internationally agreed screening cut-off.

†Growth monitoring is not practised in the respondent’s country.

### Follow-up actions

**Figure 2:**
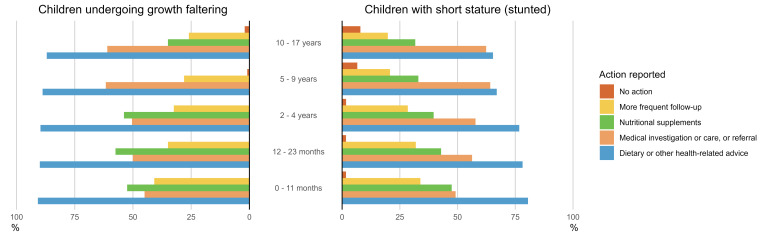
The actions taken by health service providers, in addition to counselling the parents, if a child is undergoing growth faltering (on the left) or the child has a short stature (is stunted) (on the right), % of responses. The respondents were asked to choose a maximum of three alternatives. The results are reported by child age groups.

Respondents were asked to report one to three actions taken (besides general counselling of the parents) when a child showed signs of growth faltering (*e.g.* slow weight gain or declining WAZ or LAZ). The most frequently reported action across all child age groups was the provision of nutritional or health-related advice, cited by 91% of respondents for children aged <1 year, 90% for those aged 12–23 months and 2–4 years, 89% for children aged 5–9 years, and 87% for 10–17-year-olds. The next most common actions were medical interventions, such as clinical investigation or referral for further investigation and management, and the provision of nutritional supplements with macronutrients and energy, or micronutrients. More frequent follow-up was least often reported (**Figure 2**).

With increasing child age, the proportion of respondents identifying nutritional intervention as the recommended action decreased, while the proportion recommending health interventions increased.

For a stunted child, identified as having a low attained LAZ (short stature), provision of nutritional or health advice was similarly the most frequently reported action, cited by 81% of respondents for children aged <1 year, 78% for those aged 12–23 months, 77% for children aged 2–4 years, 67% for 5–9-year-olds, and 65% for 10–17-year-olds. The next most common action taken was a health intervention, where a nutritional intervention was recommended by 47% of respondents for children aged <1 year. The proportion was smaller for older children, but still over 30% even for 10–17-year-old children. With increasing child age, the proportion of respondents identifying more frequent follow-up as the recommended action decreased, whilst the proportion recommending no further action increased. (**Figure 2**).

### Thematic analysis results

We received 58 responses through open-ended questions which asked respondents to provide any final comments or recommendations on growth monitoring. Of these, we excluded 23 from the analysis because of their general nature (*e.g.* thank you comments). The remaining 35 responses highlighted various country-specific experiences, barriers, and recommendations for improving growth monitoring systems under four thematic areas – digitalisation and data collection (particularly in Asia), standardisation and guidelines (responses evenly from all regions), barriers to effective growth monitoring, including those related to capacity building and training (particularly in Africa), and emerging issues in growth monitoring (particularly in Asia and Africa).

First, respondents reported that countries are transitioning towards digitalised growth monitoring systems, but that progress is uneven. Data collection remained inconsistent, with some countries conducting routine monitoring and others integrating it with immunisation programmes. The experts highlighted the need for easier measurement methods, improved documentation, and enhanced monitoring in primary healthcare facilities to improve data quality. Second, there was a call for enhanced standardisation. Many countries have adopted WHO growth charts, but concerns remain about their applicability across populations. The lack of standardised global monitoring tools was considered a potential source of discrepancies in data collection and interpretation.

Third, several respondents reported barriers to implementing growth monitoring, such as a lack of structured well-child visits, which led to irregular monitoring. Socioeconomic factors, including parental education, have been reported to influence attendance at growth monitoring sessions. In some regions, conflicts and economic instability exacerbated malnutrition and hindered growth monitoring efforts. Training for health personnel was often viewed as insufficient, with gaps in equipment, financing, and motivation. The integration of growth monitoring into existing health worker training curricula was recommended, but scaling up was highlighted as a challenge. There was a felt need for formative supervision and continuous professional development.

Finally, the experts identified emerging issues in growth monitoring, including pandemics, crises, and changing food and eating patterns across the globe. One respondent identified an increasing need to detect overweight and obesity, particularly in South Asia, and expressed concern that current WHO growth charts may underdiagnose overweight while over diagnosing undernutrition. They suggested incorporating additional anthropometric measures, such as the waist circumference-to-height ratio, to improve screening for metabolic health risks.

## DISCUSSION

We examined the current state of childhood growth monitoring across countries by assessing national policies, recommended practices, and follow-up actions. Drawing on responses from 122 countries, we found that, while growth monitoring is widely practised and 88% of countries have national guidance, considerable variability exists in what is measured, how often, and what actions follow growth faltering. Weight was the most commonly monitored indicator, especially in early childhood. The WHO Child Growth Standards were the most widely used reference for children under five, and the most common response to growth faltering was providing nutritional or health advice. However, the frequency and type of interventions tended to decline with child age.

Our findings align with earlier research indicating continued reliance on weight-for-age and limited integration of linear growth or growth-velocity metrics into standard monitoring [8,14,21]. Compared with data from 2000 [16], our results indicate notable progress in length/height measurements, particularly in Africa, where approximately half of children aged <5 years are now measured, up from 9% in 2000. However, many countries still rely heavily on attained size rather than growth trajectory, potentially missing early warning signs of malnutrition [8]. Similar debates regarding the applicability of WHO Child Growth Standards across diverse populations persist [12], and the growing concern over overweight and obesity [22−24], as noted in our thematic analysis, reflects emerging priorities previously underemphasised in global guidelines.

Our results show that most countries are conducting regular anthropometric measurements and utilising standardised tools, including the WHO Child Growth Standards, for childhood growth monitoring. The widespread adoption suggests a shared global commitment to improving child health. However, variability in tools, reference standards, and follow-up protocols, as observed also in other studies [25] may hinder effectiveness. Uneven application of indicators across age groups and limited use of growth velocity measures mean that in many cases, monitoring remains simplistic or reactive. This reduces its value for early detection and timely intervention. Better alignment with current scientific understanding, enhanced training, digital tools, and improved response protocols are needed to maximise impact.

Importantly, length/height measurement is increasingly recommended at least in formal guidance. In the past, the use of length measurement in growth monitoring has been debated. Some advocate its use across all age groups [1,2], while others raise concerns about the epidemiological foundations of growth, logistics, cost, and accuracy [3,26]. Yet accurate length/height measurements are essential for calculating HAZ/LAZ, WHZ/WLZ, and BMI, which are critical for identifying stunting, wasting, and overweight in children.

A concern arises in the use of nutritional interventions for children identified as having short stature or low HAZ-scores. Although this approach is common, it may be of limited benefit. In low-income settings, stunting is rarely reversed through nutritional supplementation alone [27]. Stunting is a multifactorial condition associated with infections, poor sanitation, inadequate caregiving, and environmental exposures. Therefore, offering food-based interventions alone may miss the root causes and yield little impact [3]. High prevalence of short stature can serve as a population-level warning sign of broader systemic issues, but for individual children, attained height is a poor diagnostic indicator and an unreliable target for nutritional rehabilitation [3].

The study’s cross-sectional survey approach provided a broad overview of growth monitoring practices, but had inherent limitations. Notably, allowing only one or two responses per country may not have fully captured intra-country diversity in practices. Furthermore, respondents’ knowledge and institutional affiliations may have influenced their answers. We prioritised government responses where available, which may have favoured policy-level descriptions over ground-level realities. Despite this, the overlap in answers from dual-respondent countries suggests a reasonable level of reliability. Taken together, our findings suggest that while growth monitoring is nearly universal, its operationalisation may be inconsistent and insufficient to detect or respond effectively to early growth issues.

The under-representation of certain regions, especially Oceania and parts of Europe, may limit generalisability. Furthermore, while the survey captured recommended follow-up actions, it did not explore their quality or outcomes. Policy and practice descriptions do not necessarily reflect what occurs in routine care. Finally, the lack of consensus on what constitutes growth faltering and how to address it continues to hamper efforts at standardisation and evaluation. Despite these limitations, high-level overviews such as the present study can provide critical input for future policy and guidelines updates.

## CONCLUSIONS

This study underscores both the widespread adoption of childhood growth monitoring and the significant heterogeneity in its implementation. Strengthening this practice will require global efforts to standardise indicators, promote comprehensive guidelines, and more effectively integrate growth monitoring into primary care. Through referrals and the provision of complementary services, growth monitoring can serve as an important mechanism for improving overall care and well-being. National and international stakeholders should ensure that measurement leads to action, and that action is evidence-based and equitable across age groups. As countries face evolving nutritional challenges, refining growth monitoring practices will be crucial for safeguarding child health and development globally.

## Additional material


Online Supplementary Document
