# Supplementary material for: Global assessment of childhood growth monitoring: cross-sectional survey of national policies and practices
Source: J Glob Health. 2026 Feb 6;16:04034. doi: 10.7189/jogh.16.04034 (PMC12878480; doi:10.7189/jogh.16.04034)
Supplement: Online Supplementary Document [file jogh-16-04034-s001.pdf]

Supplement to: Koivu A, Ashorn U, Borghi E, Hasman A, Menon P, Pulungan A, Ruel-Bergeron J, Shaker-Berbari L, Singh M, Thacker N, Were WM, Ylikruuvi K, Ashorn P. Global assessment of childhood growth monitoring: cross-sectional survey of national policies and practices. J Glob Health. 2026;16:04034.

Appendix S1 in the **Online Supplementary Document.** A questionnaire on national growth monitoring plans and implementation.

Section A: Questions on the target country or area.

1. What country are you reporting on?

**If you don't find the name of your country or area, choose "Other" in the drop-down menu and add the information in Q1b.*

Afghanistan
Albania
Algeria
Andorra
Angola
Antigua and Barbuda
Argentina
Armenia
Australia
Austria
Azerbaijan
Bahamas
Bahrain
Bangladesh
Barbados
Belarus
Belgium
Belize
Benin
Bhutan
Bolivia
Bosnia and Herzegovina
Botswana
Brazil
Brunei
Bulgaria
Burkina Faso
Burundi
Côte d'Ivoire
Cabo Verde
Cambodia
Cameroon
Canada
Central African Republic
Chad
Chile
China
Colombia
Comoros
Congo (Congo-Brazzaville)
Costa Rica
Croatia
Cuba
Cyprus
Czechia (Czech Republic)
Democratic Republic of the Congo

Denmark
Djibouti
Dominica
Dominican Republic
Ecuador
Egypt
El Salvador
Equatorial Guinea
Eritrea
Estonia
Eswatini (fmr. "Swaziland")
Ethiopia
Fiji
Finland
France
Gabon
Gambia
Georgia
Germany
Ghana
Greece
Grenada
Guatemala
Guinea
Guinea-Bissau
Guyana
Haiti
Holy See
Honduras
Hungary
Iceland
India
Indonesia
Iran
Iraq
Ireland
Israel
Italy
Jamaica
Japan
Jordan
Kazakhstan
Kenya
Kiribati
Kuwait
Kyrgyzstan
Laos
Latvia
Lebanon
Lesotho
Liberia
Libya
Liechtenstein
Lithuania
Luxembourg
Madagascar
Malawi

Malaysia
Maldives
Mali
Malta
Marshall Islands
Mauritania
Mauritius
Mexico
Micronesia
Moldova
Monaco
Mongolia
Montenegro
Morocco
Mozambique
Myanmar (formerly Burma)
Namibia
Nauru
Nepal
Netherlands
New Zealand
Nicaragua
Niger
Nigeria
Democratic People's Republic of Korea
North Macedonia
Norway
Oman
Pakistan
Palau
Palestine State
Panama
Papua New Guinea
Paraguay
Peru
Philippines
Poland
Portugal
Qatar
Romania
Russia
Rwanda
Saint Kitts and Nevis
Saint Lucia
Saint Vincent and the Grenadines
Samoa
San Marino
Sao Tome and Principe
Saudi Arabia
Senegal
Serbia
Seychelles
Sierra Leone
Singapore
Slovakia
Slovenia
Solomon Islands

Somalia
South Africa
South Korea
South Sudan
Spain
Sri Lanka
Sudan
Suriname
Sweden
Switzerland
Syria
Tajikistan
Tanzania
Thailand
Timor-Leste
Togo
Tonga
Trinidad and Tobago
Tunisia
Turkey
Turkmenistan
Tuvalu
Uganda
Ukraine
United Arab Emirates
United Kingdom
United States of America
Uruguay
Uzbekistan
Vanuatu
Venezuela
Vietnam
Yemen
Zambia
Zimbabwe
Other

Q1b. If other, please specify.

Section B: Questions on national guidance, purpose and sites of childhood growth monitoring

2. To your knowledge, is there a national guidance or other document that provides instructions on the implementation of growth monitoring in childhood in your country?

a. Yes, there is guidance issued by one organization / authority
b. Yes, there is guidance issued by two or more separate organizations / authorities
c. No
d. Don't know

2b. If yes, who has issued it or them (multiple options possible, if previous answer was (b)

a. The national ministry responsible for child health, public health, or nutrition
b. A national pediatric society or association
c. Another party. Please specify.
d. Don't know
e. Not applicable, there is no guidance document on growth monitoring

2c.If another Party, Please specify.

3. Is the purpose of growth monitoring stated in the guidance document that you refer to in questions 2 and 2b?

a. Yes, explicitly
b. Yes, but not explicitly
c. No
d. Don't know
e. Not applicable (I am not aware of any national guidance document of growth monitoring)

4. What are the purposes* of growth monitoring in your country ? (choose all that apply)

** This question assumes that growth monitoring is an activity that has some other purpose besides neutrally documenting a change in the size of a child. In your response, consider these purposes - why is children's growth monitored in your country. Base your answer on the relevant guidance document, if the purpose is mentioned there. If it is not mentioned or there is no guidance document, base the answer on your own opinion.*

***When growth deviates, there can be an underlying health or nutritional problem - so the question is about the deviations that are being sought with growth monitoring. When the child grows "normally", the parents can usually be assured that the child is healthy and in a good nutritional status*

a. Identification of insufficient intake of energy and nutrients (typically presenting as severe weight loss or having low or very low weight-for-height, MUAC, or BMI = being wasted)
b. Identification of excess intake of energy and nutrients (typically presenting as rapid weight gain or high or very high weight-for-height or BMI = being overweight or obese))
c. Identification of chronic illnesses (presenting as linear growth faltering or having low length-for-age or low height-for-age =being stunted)
d. Identification of genetic growth-affecting conditions (can present as decelerated or accelerated linear growth or weight gain)
e. Identification of an imminent or existing food emergency (indicated by a large proportion of children being wasted, i.e. having very low weight-for-height or MUAC)
f. Identification of an adversity of children's growth environment (indicated by a large proportion of children being stunted, i.e. having low length-for-age or height-for-age)
g. Something else. Please specify.
h. Don't know
i. Not applicable, growth monitoring is not practiced in my country

4a.If something else. Please specify.

5. In your opinion, which of these are the three most important purposes of growth monitoring in your country?

(This question assumes that growth monitoring is an activity that has some other purpose than just neutrally documenting a change in the size of a child. In your response, consider these purposes - why is children's growth monitored in your country. Mark 1 for the most important purpose, 2 for the second most important and 3 for the third most important purpose)

a. Identification of insufficient intake of energy and nutrients
b. Identification of excess intake of energy and nutrients
c. Identification of chronic illnesses
d. Identification of genetic growth-affecting conditions
e. Identification of an imminent or existing food emergency
f. Identification of an adversity of children's growth environment
g. Something else
h. Don't know
i. Not applicable, growth monitoring is not practiced in my country

6. Where is growth monitoring conducted in your country?

(check all that apply)

a. At well-child visits* at public health facilities
b. At well-child visits at private health facilities
c. At kindergartens, preschools and schools
d. As part of facility-based nutrition programs
e. As part of outreach nutrition programs
f. During vaccination campaigns
g. At outpatient clinics treating ill patients
h. Somewhere else, Please specify:
i. Don't know.
j. Not applicable, growth monitoring is not practiced in my country

6a.If somewhere else, Please specify.

7. In which of the previous settings is growth monitoring most conducted in your country?

a. At well-child visits at public health facilities
b. At well-child visits at private health facilities
c. At kindergartens, preschools and schools
d. As part of facility-based nutrition programs
e. As part of outreach nutrition programs
f. During vaccination campaigns
g. At outpatient clinics treating ill patients
h. Somewhere else, Please specify.
i. Don't know.
j. Not applicable, growth monitoring is not practiced in my country

7a. If somewhere else, please specify.

Section C: Questions on the contents of growth monitoring at well-child visits. All the questions in section C concern primarily growth monitoring conducted at well-child visits. If there are no well-child visits in your country, please answer section C questions in relation to the setting that you chose as the main growth monitoring site in the previous question 7.

8. For each of the following age intervals, how many times do children undergo growth monitoring as part of well-child visits in your country?

Please provide two answers for each age-group. Indicate the nationally recommended number of visits (per child) in the first column and your estimate on actual mean number per child in the second column.*

**The number recommended by WHO and UNICEF is provided in parentheses.*

***Mark 99 if you don't know a specific number, mark 88 if the question is not applicable, i.e. growth monitoring is not conducted at well-child visits in your country.*

****In case age categorization is different in your country, you can provide further information in question 8(I).*

	Age interval	Nationally recommended number of visits	Average number of actual visits
8a.	0 -11 month-old children (8**)		
8b.	12-23 month-old children (2)		
8c.	2-4 year-old children (3)		
8d.	5-9 year-old children (2)		
8e.	10-17 year-old children (2)		

8(I). Free comments

In the following questions(q9a-9e), we ask your opinion on how commonly selected anthropometric measurements are taken from children in various age intervals. The five questions are otherwise identical, but each of them concerns a different age group. For the setting, please refer to instructions at the top of this page, above question 8.

9a. In your opinion, how commonly are the following anthropometric measurements taken during growth monitoring of 0-11 month-old children* ?

	Always	Almost always	Sometimes	Never	Don't know	Not applicable
9a(i). Weight						
9a(ii). Length						
9a(iii). Head circumference						
9a(iv). Mid upper arm circumference (MUAC)						
9a(v). Some other measures						

9av(i). If some other measures are taken during growth monitoring of 0-11 month-old children, Please specify.

9b. In your opinion, how commonly are the following anthropometric measurements taken during growth monitoring of 12-23 month-old children* ?

	Always	Almost always	Sometimes	Never	Don't know	Not applicable
9b(i). Weight						
9b(ii). Length						
9b(iii). Head circumference						
9b(iv). Mid upper arm circumference (MUAC)						
9b(v). Some other measures						

9bv(i). If some other measures are taken during growth monitoring of 12-23 month-old children, Please specify.

9c. In your opinion, how commonly are the following anthropometric measurements taken during growth monitoring of 2-4 year-old children* ?

	Always	Almost always	Sometimes	Never	Don't know	Not applicable
9c(i). Weight						
9c(ii). Length						
9c(iii). Head circumference						
9c(iv). Mid upper arm circumference (MUAC)						
9c(v). Some other measures						

9cv(i). If some other measures are taken during growth monitoring of 2-4 year-old children, Please specify.

9d. In your opinion, how commonly are the following anthropometric measurements taken during growth monitoring of 5-9 year-old children* ?

	Always	Almost always	Sometimes	Never	Don't know	Not applicable
9d(i). Weight						
9d(ii). Length						
9d(iii). Head circumference						
9d(iv). Mid upper arm circumference (MUAC)						
9d(v). Some other measures						

9dv(i). If some other measures are taken during growth monitoring of 5-9 year-old children, Please specify.

9e. In your opinion, how commonly are the following anthropometric measurements taken during growth monitoring of 10-17 year-old children* ?

	Always	Almost always	Sometimes	Never	Don't know	Not applicable
9e(i). Weight						
9e(ii). Length						
9e(iii). Head circumference						
9e(iv). Mid upper arm circumference (MUAC)						
9e(v). Some other measures						

9ev(i). If some other measures are taken during growth monitoring of 10-17 year-old children, Please specify.

10. In the well-child visit setting, who most commonly interprets the adequacy of growth and makes decisions on possible treatment or referral?

** If there are no well-child visits in your country, consider the growth monitoring setting that you indicated in q8.*

a. A community health worker or another representative of auxiliary health staff
b. A specially trained growth monitor
c. A nurse or another health facility staff member
d. A physician / medical doctor
e. Someone else. Please specify.
f. Don't know
g. Not applicable, growth monitoring is not practiced in my country

10a. If someone else conducts the anthropometric measurements for growth monitoring in your country, Please specify.

11. Please list the anthropometric indices that are usually calculated for growth monitoring in your country (choose all that apply)

a. Weight-for-age z-score (WAZ)
b. Weight-for-length/height z-score (WLZ / WHZ)
c. Length/height for age z-score (LAZ / HAZ)
d. Body-mass index (BMI)
e. BMI z-score (BMIZ)
f. Something else. Please specify.
g. Don't know
h. Not applicable, growth monitoring is not practiced in my country

11a. If some other anthropometric indices are calculated for growth monitoring in your country, please specify.

12a. What growth reference is used for comparison and calculating these anthropometric indices for under-five-year-old children?

(choose all that apply).

a. World Health Organization Child Growth Standards
b. A national growth reference/standard.
c. Something else. Please specify.
d. Don't know.
e. Not applicable, growth monitoring is not practiced in this age group in my country.

12a(i). If something else growth reference is used, please specify.

12b. What growth reference is used for comparison and calculating these anthropometric indices for over-five-year-old children?

(choose all that apply)

a. World Health Organization Child Growth Standards
b. A national growth reference/standard.
c. Something else. Please specficy.
d. Don't know.
e. Not applicable, growth monitoring is not practiced in this age group in my country

12b(i). If something else growth reference is used, please specify.

13. How are the anthropometric indices calculated?. Choose the most common option.

a. Plotting the weight and/or height values in a sex-specific growth chart on paper
b. Using a paper-based table that converts raw weight and/or height values into relative values (Z-scores)
c. Using a specific digital software (WHO Anthro or another software)
d. Entering the raw age, sex, weight, and height values into a growth monitoring software that calculates indices and plots the growth curves graphically.
e. Something else. Please specify.
f. Don't know
g. Not applicable, growth monitoring is not practiced in my country

13a. If something else, please specify.

Section D: Questions on result interpretation and follow-up actions

14. In the well-child visit setting, who most commonly conducts the anthropometric measurements for growth monitoring in your country?

**If there are no well-child visits in your country, consider the growth monitoring setting that you indicated in q8.*

a. A community health worker or another representative of auxiliary health staff
b. A specially trained growth monitor
c. A nurse or another health facility staff member
d. A physician / medical doctor
e. Someone else. Please specify.
f. Don't know
g. Not applicable, growth monitoring is not practiced in my country

14a. If something else, Please specify.

15. What criteria are used to determine if there is a problem with the child's growth*?

(choose all that apply)

**A value that is below and above a nationally or internationally agreed screening cut-off.*

WAZ = weight-for-age Z-score, WLZ = weight-for-length Z-score, WHZ = weight-for-height Z-score, LAZ = length-for-age Z-score, HAZ = height-for-age Z-score, MUAC = Mid upper arm circumference

a. Too low or too high* value in the latest WAZ-score (attained WAZ)
b. Too low or too high value in the latest WLZ or WHZ-score (attained WLZ or WHZ)
c. Too low or too high value in the latest MUAC-value (attained MUAC)
d. Too low or too high value in the latest LAZ or HAZ-value (attained LAZ or HAZ)
e. Too low or too high value in parental-height adjusted LAZ or HAZ
f. Too low or too high value in in the change in WHZ between different measurements (too low or too high velocity in weight gain)
g. Too low or too high value in the change in LAZ / HAZ between different measurements (too low or too high velocity in length / height gain)
h. Something else. Please specify.
i. Don't know
j. Not applicable, growth monitoring is not practiced in my country

15a. If something else, Please specify.

In the following questions (q16a-q16e), we ask your opinion about the most common actions that are taken if a child is undergoing growth faltering in various age intervals. The five questions are otherwise identical, but each of them concerns a different age group.

16a. What are the 1-3 most common actions*in addition to counselling the parents, if a child is undergoing growth faltering at 0-11 months?

Choose a maximum of 3 alternatives.

**Growth faltering means that the child is growing slower than expected. i.e.,S/he has slow weight gain or decreasing Z-score in weight-for-age (WAZ) or length for age (LAZ).*

a. Nothing
b. Provision of advice on adequate diet
c. Provision of other health-related advice
d. Provision of nutritional supplements with macronutrients and energy
e. Provision of micronutrient supplements (minerals and vitamins)
f. More frequent follow-up at the growth monitoring clinic
g. A home visit
h. Further medically investigations (locally)
i. Provision of medical care (locally)
j. Referral for further investigations and management
k. Something else. Please Specify.
l. Don't know
m. No applicable, this condition would not be detected in this age group

16a(i). If something else, please specify.

16b. What are the 1-3 most common actions*in addition to counselling the parents, if a child is undergoing growth faltering at 12-23 months?

Choose a maximum of 3 alternatives.

**Growth faltering means that the child is growing slower than expected. i.e.,S/he has slow weight gain or decreasing Z-score in weight-for-age (WAZ) or length for age (LAZ).*

a. Nothing
b. Provision of advice on adequate diet
c. Provision of other health-related advice
d. Provision of nutritional supplements with macronutrients and energy
e. Provision of micronutrient supplements (minerals and vitamins)
f. More frequent follow-up at the growth monitoring clinic
g. A home visit
h. Further medically investigations (locally)
i. Provision of medical care (locally)
j. Referral for further investigations and management
1k. Something else. Please Specify.
l. Don't know
m. No applicable, this condition would not be detected in this age group

16b(i). If something else, please specify.

16c. What are the 1-3 most common actions*in addition to counselling the parents, if a child is undergoing growth faltering at 2-4 years?

Choose a maximum of 3 alternatives.

**Growth faltering means that the child is growing slower than expected. i.e.,S/he has slow weight gain or decreasing Z-score in weight-for-age (WAZ) or length for age (LAZ).*

a. Nothing
b. Provision of advice on adequate diet
c. Provision of other health-related advice
d. Provision of nutritional supplements with macronutrients and energy
e. Provision of micronutrient supplements (minerals and vitamins)
f. More frequent follow-up at the growth monitoring clinic
g. A home visit
h. Further medically investigations (locally)
i. Provision of medical care (locally)
j. Referral for further investigations and management
k. Something else. Please Specify.
l. Don't know
m. No applicable, this condition would not be detected in this age group

16c(i). If something else, please specify.

16d. What are the 1-3 most common actions*in addition to counselling the parents, if a child is undergoing growth faltering at 5-9 years?

Choose a maximum of 3 alternatives.

**Growth faltering means that the child is growing slower than expected. i.e.,S/he has slow weight gain or decreasing Z-score in weight-for-age (WAZ) or length for age (LAZ).*

a. Nothing
b. Provision of advice on adequate diet
c. Provision of other health-related advice
d. Provision of nutritional supplements with macronutrients and energy
e. Provision of micronutrient supplements (minerals and vitamins)
f. More frequent follow-up at the growth monitoring clinic
g. A home visit
h. Further medically investigations (locally)
i. Provision of medical care (locally)
j. Referral for further investigations and management
k. Something else. Please Specify.
l. Don't know
m. No applicable, this condition would not be detected in this age group

16d(i). If something else, please specify.

16e. What are the 1-3 most common actions*in addition to counselling the parents, if a child is undergoing growth faltering at 10-17 years?

Choose a maximum of 3 alternatives.

**Growth faltering means that the child is growing slower than expected. i.e.,S/he has slow weight gain or decreasing Z-score in weight-for-age (WAZ) or length for age (LAZ).*

a. Nothing
b. Provision of advice on adequate diet
c. Provision of other health-related advice
d. Provision of nutritional supplements with macronutrients and energy
e. Provision of micronutrient supplements (minerals and vitamins)
f. More frequent follow-up at the growth monitoring clinic
g. A home visit
h. Further medically investigations (locally)
i. Provision of medical care (locally)
j. Referral for further investigations and management
k. Something else. Please Specify.
l. Don't know
m. No applicable, this condition would not be detected in this age group

16e(i). If something else, please specify.

In the following questions (q17a-17e), we ask your opinion about the most common actions that are taken if a child is undergoing as stunted in various age intervals. The five questions are otherwise identical, but each of them concerns a different age group.

The difference between q16 and q17 is that in q16 we ask about the growth velocity, i.e., the child is identified as growing slower than expected (noted by a declining WAZ or HAZ score), whereas in q17 we ask about attained (current length or height, i.e. the child is identified as being stunted (short-for-age, noted by a LAZ or WAZ score that is below -2).

17a. What are the 1-3 most common actions* in addition to counselling the parents, if a child is identified as stunted at the age-group 0 -11 months?

Choose a maximum of 3 alternatives.

**Being stunted means that the child has low length-for-age Z score (LAZ).*

a. Nothing
b. Provision of advice on adequate diet
c. Provision of other health-related advice
d. Provision of nutritional supplements with macronutrients and energy
e. Provision of micronutrient supplements (minerals and vitamins)
f. More frequent follow-up at the growth monitoring clinic
g. A home visit
h. Further medically investigations (locally)
i. Provision of medical care (locally)
j. Referral for further investigations and management

k. Something else. Please Specify.
l. Don't know
m. No applicable, this condition would not be detected in this age group

17a(i). If something else, please specify.

17b. What are the 1-3 most common actions* in addition to counselling the parents, if a child is identified as stunted at the age-group 12-23 months?

Choose a maximum of 3 alternatives.

**Being stunted means that the child has low length-for-age Z score (LAZ).*

a. Nothing
b. Provision of advice on adequate diet
c. Provision of other health-related advice
d. Provision of nutritional supplements with macronutrients and energy
e. Provision of micronutrient supplements (minerals and vitamins)
f. More frequent follow-up at the growth monitoring clinic
g. A home visit
h. Further medically investigations (locally)
i. Provision of medical care (locally)
j. Referral for further investigations and management
k. Something else. Please Specify.
l. Don't know
m. No applicable, this condition would not be detected in this age group

17b(i). If something else, please specify.

17c. What are the 1-3 most common actions* in addition to counselling the parents, if a child is identified as stunted at the age-group 2-4 years?

Choose a maximum of 3 alternatives.

**Being stunted means that the child has low length-for-age Z score (LAZ).*

a. Nothing
b. Provision of advice on adequate diet
c. Provision of other health-related advice
d. Provision of nutritional supplements with macronutrients and energy
e. Provision of micronutrient supplements (minerals and vitamins)
f. More frequent follow-up at the growth monitoring clinic
g. A home visit
h. Further medically investigations (locally)
i. Provision of medical care (locally)
j. Referral for further investigations and management
k. Something else. Please Specify.
l. Don't know
m. No applicable, this condition would not be detected in this age group

17c(i). If something else, please specify.

17d. What are the 1-3 most common actions* in addition to counselling the parents, if a child is identified as stunted at the age-group 5-9 years?

Choose a maximum of 3 alternatives.

**Being stunted means that the child has low length-for-age Z score (LAZ).*

a. Nothing
b. Provision of advice on adequate diet
c. Provision of other health-related advice
d. Provision of nutritional supplements with macronutrients and energy
e. Provision of micronutrient supplements (minerals and vitamins)
f. More frequent follow-up at the growth monitoring clinic
g. A home visit
h. Further medically investigations (locally)
i. Provision of medical care (locally)
j. Referral for further investigations and management
k. Something else. Please Specify.
l. Don't know
m. No applicable, this condition would not be detected in this age group

17d(i). If something else, please specify.

17e. What are the 1-3 most common actions* in addition to counselling the parents, if a child is identified as stunted at the age-group 10-17 years?

Choose a maximum of 3 alternatives.

**Being stunted means that the child has low length-for-age Z score (LAZ).*

a. Nothing
b. Provision of advice on adequate diet
c. Provision of other health-related advice
d. Provision of nutritional supplements with macronutrients and energy
e. Provision of micronutrient supplements (minerals and vitamins)
f. More frequent follow-up at the growth monitoring clinic
g. A home visit
h. Further medically investigations (locally)
i. Provision of medical care (locally)
j. Referral for further investigations and management
k. Something else. Please Specify.
l. Don't know
m. No applicable, this condition would not be detected in this age group

17e(i). If something else, please specify.

Section E: Questions on staff training, quality assurance, and administrative use of data

18. According to your information, which of the following training activities on growth monitoring take place in your country?

(choose all that apply)

a. Theoretical lectures for nursing students
b. Hands on training for nursing students
c. Refresher training for nurses conducting growth monitoring
d. Theoretical lectures for medical students
e. Hands on training for medical students
f. Refresher training for physicians conducting growth monitoring
g. Theoretical lectures for other students
h. Hands on training for other students
i. Refresher training for other staff conducting growth monitoring
j. None of the above
k. Don't know

19. To your knowledge, what proportion of growth monitoring sites undergo the following quality assurance or quality control measures?

For each question, choose a value between 0% (none) and 100% (all sites).

If you don't have an estimate for a particular activity, click on the "Don't know or not applicable" button on the right of the scale.

19a. Regular calibration of scales with standard weights

19b. Regular calibration of scales with standard weights

19c. Regular calibration of length boards and stadiometres with standard length calibrators

19d. Regular calibration of length boards and stadiometres with standard length calibrators

19e. Regular calibration of head and arm circumference tapes with standard length calibrators

19f. Regular calibration of head and arm circumference tapes with standard length calibrators

19g. Regular quality checks for accurate measuring techniques

19h. Regular quality checks for accurate measuring techniques

19i. Regular analysis and feedback on the coverage of growth monitoring

19j. Regular analysis and feedback on the coverage of growth monitoring

20. Where are the anthropometric results usually recorded?

(choose all that apply)

a. Home-based under-five-cards
b. Other home-based records
c. Facility-based paper records
d. Facility-based digital records

e. Digital records that are available nationally to health personnel
f. Digital records that are available to the child and her family
g. Somewhere else. Please specify.
h. Not applicable, growth monitoring is not practiced in my country
i. Don't know

20a. If somewhere else, please specify.

21. Are growth monitoring results reported and summarized through administrative data systems?

a. Yes, systematically
b. Yes, but not systematically
c. No
d. Not applicable, growth monitoring is not practiced in my country
e. Don't know

22. What proportion of children do you estimate are covered by growth monitoring at well-child visits in your country?

Primarily based on administrative data. If that is not available, respondents estimate.

	0-25%	26-50%	51-75%	76-100%	Don't know	Not applicable
22a. 0-11 months						
22b. 12-23 months						
22c. 2-4 years						
22d. 5-9 years						
22e. 10-17 years						

Section F: Questions about the respondent

23. In what capacity are you responding?

a. A representative of a national ministry or another national institution with an oversight on growth monitoring
b. A representative of a national paediatric society
c. Another party (specify).

23a. If the option is (a),
(i) Which ministry or institution
(ii) Which department or unit?

23b. If the option is another party, please specify.

24. Do you wish to be included in an author group in a publication on the survey results?

Yes
No

24a. If yes, please provide further details.
1. Your name
2. Your affiliation (organization & country)
3. Your email address

25. Any free comments from you to the growth monitoring survey team:

Appendix S2 in the **Online Supplementary Document.**

There are 29 countries from which there is a survey response from the ministry/UNICEF and the national padiatric association. Countries have been given pseudonyms C1–C29 and they are in random order in the following tables.

2. To your knowledge, is there a national guidance or other document that provides instructions on the implementation of growth monitoring in childhood in your country?

**Country**	**Ministry**	**IPA**
C1	a. Yes, there is guidance issued by one organization / authority	a. Yes, there is guidance issued by one organization / authority
C2	a. Yes, there is guidance issued by one organization / authority	b. Yes, there is guidance issued by two or more separate organizations / authorities
C3	a. Yes, there is guidance issued by one organization / authority	a. Yes, there is guidance issued by one organization / authority
C4	a. Yes, there is guidance issued by one organization / authority	
C5	a. Yes, there is guidance issued by one organization / authority	a. Yes, there is guidance issued by one organization / authority
C6	a. Yes, there is guidance issued by one organization / authority	a. Yes, there is guidance issued by one organization / authority
C7	b. Yes, there is guidance issued by two or more separate organizations / authorities	a. Yes, there is guidance issued by one organization / authority
C8	a. Yes, there is guidance issued by one organization / authority	b. Yes, there is guidance issued by two or more separate organizations / authorities
C9	a. Yes, there is guidance issued by one organization / authority	b. Yes, there is guidance issued by two or more separate organizations / authorities
C10	a. Yes, there is guidance issued by one organization / authority	b. Yes, there is guidance issued by two or more separate organizations / authorities
C11	a. Yes, there is guidance issued by one organization / authority	b. Yes, there is guidance issued by two or more separate organizations / authorities
C12	a. Yes, there is guidance issued by one organization / authority	a. Yes, there is guidance issued by one organization / authority
C13	a. Yes, there is guidance issued by one organization / authority	a. Yes, there is guidance issued by one organization / authority

Country	Ministry	IPA
C14	b. Yes, there is guidance issued by two or more separate organizations / authorities	b. Yes, there is guidance issued by two or more separate organizations / authorities
C15	a. Yes, there is guidance issued by one organization / authority	a. Yes, there is guidance issued by one organization / authority
C16	a. Yes, there is guidance issued by one organization / authority	b. Yes, there is guidance issued by two or more separate organizations / authorities
C17	a. Yes, there is guidance issued by one organization / authority	a. Yes, there is guidance issued by one organization / authority
C18	c. No	b. Yes, there is guidance issued by two or more separate organizations / authorities
C19	a. Yes, there is guidance issued by one organization / authority	a. Yes, there is guidance issued by one organization / authority
C20	a. Yes, there is guidance issued by one organization / authority	a. Yes, there is guidance issued by one organization / authority
C21	a. Yes, there is guidance issued by one organization / authority	b. Yes, there is guidance issued by two or more separate organizations / authorities
C22	a. Yes, there is guidance issued by one organization / authority	a. Yes, there is guidance issued by one organization / authority
C23	a. Yes, there is guidance issued by one organization / authority	b. Yes, there is guidance issued by two or more separate organizations / authorities
C24	a. Yes, there is guidance issued by one organization / authority	c. No
C25	a. Yes, there is guidance issued by one organization / authority	a. Yes, there is guidance issued by one organization / authority
C26	a. Yes, there is guidance issued by one organization / authority	a. Yes, there is guidance issued by one organization / authority
C27	a. Yes, there is guidance issued by one organization / authority	a. Yes, there is guidance issued by one organization / authority
C28	a. Yes, there is guidance issued by one organization / authority	a. Yes, there is guidance issued by one organization / authority
C29	a. Yes, there is guidance issued by one organization / authority	b. Yes, there is guidance issued by two or more separate organizations / authorities

2b. If yes, who has issued it or them (multiple options possible, if previous answer was (b))

	National ministry		National pediatric society		Other	
**Country**	**Ministry**	**IPA**	**Ministry**	**IPA**	**Ministry**	**IPA**
C1	X	X				
C2	X	X	X	X	X	
C3	X	X				
C4	X					
C5	X			X		
C6	X					
C7		X	X			
C8		X			X	X
C9	X	X		X		
C10	X	X				
C11	X	X				
C12	X	X				
C13	X	X				
C14			X	X	X	
C15	X	X				
C16	X	X				X
C17	X					
C18		X				
C19	X	X				
C20	X	X				
C21	X	X				
C22	X	X				
C23	X	X		X		
C24	X					
C25	X	X				
C26	X	X				
C27	X	X				
C28	X	X				
C29	X	X				

9. In your opinion, how commonly are the following anthropometric measurements taken during growth monitoring? Indicate “not applicable” if growth monitoring is not practiced in this age group. MUAC = Mid upper arm circumference

9a. 0-11 month-old children

	**Weight**		**Length**		**Head circumference**		**MUAC**	
**Country**	**Ministry**	**IPA**	**Ministry**	**IPA**	**Ministry**	**IPA**	**Ministry**	**IPA**
C1	Always	Always	NA	Always	NA	Sometimes	Almost always	Always
C2	Always	Always	Always	Always	Sometimes	Sometimes	Sometimes	Sometimes
C3	Always	Always	Always	Always	Almost always	Always	Sometimes	Always
C4	Always	Always	Always	Always	Always	Always	Never	Never
C5	Always	Always	Almost always	Always	Sometimes	Almost always	Almost always	Almost always
C6	Always	Almost always	Always	Sometimes	Always	Sometimes	Never	Never
C7	Always	Always	Always	Always	Always	Always	Sometimes	Never
C8	Always	Always	Always	Sometimes	Sometimes	Never	Always	Sometimes
C9	Always	Always	Always	Sometimes	Always	Sometimes	Always	Don’t know
C10	Always	Always	Always	Almost always	Sometimes	Sometimes	Sometimes	Almost always
C11	Always	Always	Sometimes	Sometimes	Never	Never	Almost always	Never
C12	Almost always	Always	Almost always	Always	NA	Always	Sometimes	Sometimes
C13	Always	Always	Sometimes	Sometimes	Sometimes	Sometimes	Sometimes	Sometimes
C14	Always	Always	Almost always	Almost always	Almost always	Almost always	Never	Almost always
C15	Always	Always	Always	Always	Always	Always	NA	Sometimes
C16	Always	Always	Almost always	Sometimes	Almost always	Never	Almost always	Sometimes
C17	Always	Always	Sometimes	Sometimes	Always	Never	Sometimes	Sometimes
C18	Always	Always	Always	Almost always	NA	Sometimes	Sometimes	Almost always
C19	Always	Always	Sometimes	Always	Never	Sometimes	Almost always	Sometimes
C20	Always	Always	Always	Always	Always	Always	NA	Sometimes

Country	Weight		Length		Head circumference		MUAC	
	Ministry	IPA	Ministry	IPA	Ministry	IPA	Ministry	IPA
C21	Almost always	Always	Almost always	Almost always	Sometimes	Almost always	Almost always	Always
C22	Always	Always	Always	Always	Sometimes	Always	Never	NA
C23	Always	Always	Always	Always	Always	Sometimes	Always	Sometimes
C24	Sometimes	Always	Sometimes	Don't know	Sometimes	Always	Sometimes	NA
C25	Always	Almost always	Almost always	Sometimes	Never	Sometimes	Always	Almost always
C26	Always	Always	Always	Always	Always	Sometimes	Always	Always
C27	Always	Always	Almost always	Almost always	Almost always	Almost always	Sometimes	Sometimes
C28	Always	Always	Always	Always	Always	Always	Sometimes	Sometimes
C29	Always	Almost always	Always	Almost always	Always	Sometimes	Sometimes	Never

9b. 12-23 month-old children

Country	Weight		Length		Head circumference		MUAC	
	Ministry	IPA	Ministry	IPA	Ministry	IPA	Ministry	IPA
C1	Always	Always	NA	Always	NA	Sometimes	Almost always	Always
C2	Always	Always	Always	Always	Never	Never	Sometimes	Sometimes
C3	Always	Always	Always	Always	Almost always	Always	Sometimes	Always
C4	Always	Always	Always	Always	Always	Always	Never	Never
C5	Always	Always	Almost always	Always	Sometimes	Almost always	Almost always	Almost always
C6	Always	Always	Always	Almost always	Sometimes	Sometimes	Never	Never
C7	Always	Always	Always	Always	Almost always	Always	Sometimes	Never
C8	Always	Sometimes	Always	Sometimes	NA	Never	Always	Sometimes
C9	Sometimes	Always	Sometimes	Sometimes	Don't know	Sometimes	Always	Don't know
C10	Always	Always	Always	Almost always	Never	Sometimes	Sometimes	NA

Country	Weight		Length		Head circumference		MUAC	
	Ministry	IPA	Ministry	IPA	Ministry	IPA	Ministry	IPA
C11	Always	Always	Sometimes	Sometimes	Never	Never	Almost always	Never
C12	Almost always	Always	Almost always	Always	NA	Always	Sometimes	Sometimes
C13	Always	Always	Sometimes	Sometimes	Never	Sometimes	Sometimes	Sometimes
C14	Always	Always	Sometimes	Sometimes	Almost always	Sometimes	Sometimes	Sometimes
C15	Always	Always	Always	Always	Always	Sometimes	NA	Don't know
C16	Almost always	Always	Almost always	Sometimes	Never	Never	Almost always	Sometimes
C17	Always	Always	Sometimes	Almost always	Sometimes	Never	Sometimes	Don't know
C18	Always	Always	Always	Almost always	NA	Sometimes	Always	Sometimes
C19	Always	Always	Almost always	Always	Never	Sometimes	Always	Always
C20	Always	Always	Always	Always	Always	Always	NA	Sometimes
C21	Almost always	Always	Almost always	Almost always	Don't know	Almost always	Almost always	Always
C22	Always	Always	Always	Always	Sometimes	NA	Never	Always
C23	Always	Always	Always	Always	Always	Sometimes	Always	Sometimes
C24	Sometimes	Always	Sometimes	NA	Sometimes	Sometimes	Sometimes	NA
C25	Always	Almost always	Almost always	Sometimes	Never	Sometimes	Always	Almost always
C26	Always	Always	Always	Always	Always	Sometimes	Always	Always
C27	Almost always	Always	Almost always	Always	Sometimes	Almost always	Sometimes	Sometimes
C28	Always	Always	Always	Always	NA	Almost always	Sometimes	Sometimes
C29	Always	Always	Always	Never	Sometimes	Never	Sometimes	Never

9c. 2-4 year-old children

Country	Weight		Length		Head circumference		MUAC	
	Ministry	IPA	Ministry	IPA	Ministry	IPA	Ministry	IPA
C1	NA	Always	NA	Always	NA	Sometimes	NA	Always
C2	Always	Always	Always	Always	Never	Never	Sometimes	Sometimes
C3	Always	Always	Always	Always	Never	Always	Never	Sometimes
C4	Always	Always	Always	Always	Always	Sometimes	Never	Never
C5	Always	Always	Almost always	Always	Sometimes	Sometimes	Almost always	Sometimes
C6	Always	Always	Always	Almost always	Never	Sometimes	Never	Never
C7	Always	Always	Always	Always	Sometimes	Always	Sometimes	Never
C8	Always	Sometimes	Always	Sometimes	NA	Never	Always	Sometimes
C9	Sometimes	Always	Sometimes	Sometimes	Don't know	Sometimes	Always	Don't know
C10	Almost always	Always	Almost always	Almost always	Never	NA	Sometimes	NA
C11	Always	Always	Sometimes	Never	Never	Never	Almost always	Never
C12	Almost always	Always	Almost always	Always	NA	Never	Sometimes	Sometimes
C13	Always	Always	Sometimes	Sometimes	Never	Never	Sometimes	Sometimes
C14	Always	Always	Sometimes	Sometimes	Sometimes	Sometimes	Sometimes	Sometimes
C15	Always	Always	Always	Always	Almost always	NA	NA	Sometimes
C16	Almost always	Almost always	Sometimes	Sometimes	Never	Never	Almost always	Sometimes
C17	Always	Always	Sometimes	Almost always	Sometimes	Never	Sometimes	Don't know
C18	Always	Always	Always	Almost always	NA	Sometimes	Always	Sometimes
C19	Always	Always	Sometimes	Always	Never	Sometimes	Always	Always
C20	Always	Always	Always	Always	Sometimes	Sometimes	NA	Sometimes
C21	Almost always	Always	Almost always	Almost always	Never	Almost always	Almost always	Always
C22	Always	Always	Always	Always	Never	NA	Never	Always
C23	Always	Always	Always	Always	Always	Sometimes	Always	Sometimes

Country	Weight		Length		Head circumference		MUAC	
	Ministry	IPA	Ministry	IPA	Ministry	IPA	Ministry	IPA
C24	Sometimes	Always	Sometimes	NA	Sometimes	NA	Sometimes	NA
C25	NA	Sometimes	NA	Sometimes	NA	Sometimes	NA	Sometimes
C26	Always	Always	Always	Always	NA	Sometimes	Always	Always
C27	Always	Always	Always	Always	Sometimes	Sometimes	Sometimes	Sometimes
C28	Always	Always	Always	Always	Always	Sometimes	Sometimes	Sometimes
C29	Always	Almost always	Always	Never	Sometimes	Never	Sometimes	Never

9d. 5-9 year-old children

Country	Weight		Length		Head circumference		MUAC	
	Ministry	IPA	Ministry	IPA	Ministry	IPA	Ministry	IPA
C1	NA	Always	NA	Always	NA	Sometimes	NA	Always
C2	Never	Always	Never	Always	Never	Never	Never	Sometimes
C3	Always	Always	Always	Always	Never	Sometimes	Never	Sometimes
C4	Always	Always	Always	Always	Sometimes	Never	Never	Never
C5	NA	Always	NA	Always	NA	Sometimes	NA	Sometimes
C6	Always	Always	Always	Always	Never	Sometimes	Never	Never
C7	Always	Always	Always	Always	Never	Always	Sometimes	Never
C8	NA	Sometimes	NA	Sometimes	NA	Never	NA	Never
C9	Sometimes	Always	Sometimes	Almost always	NA	Never	NA	Don't know
C10	Sometimes	Always	Sometimes	Almost always	Never	NA	Never	NA
C11	NA	Never	NA	Never	NA	Never	NA	Never
C12	NA	Always	NA	Always	NA	Never	NA	Sometimes
C13	NA	NA	NA	NA	NA	NA	NA	NA
C14	Always	Always	Sometimes	Sometimes	Sometimes	Sometimes	Sometimes	Sometimes
C15	Always	Always	Always	Always	Never	NA	NA	NA
C16	Sometimes	Almost always	Sometimes	Sometimes	Never	Never	Never	Never
C17	Sometimes	Always	Sometimes	Don't know	NA	Don't know	NA	Don't know

Country	Weight		Length		Head circumference		MUAC	
	Ministry	IPA	Ministry	IPA	Ministry	IPA	Ministry	IPA
C18	Sometimes	Always	Sometimes	Almost always	NA	Sometimes	Sometimes	Sometimes
C19	Never	Always	Never	Always	Never	Never	Never	Always
C20	Always	Always	Always	Always	Don't know	Never	NA	Sometimes
C21	Sometimes	Sometimes	Sometimes	Sometimes	NA	Don't know	NA	Sometimes
C22	Almost always	Always	Almost always	Always	Never	NA	Never	Sometimes
C23	Always	Always	Always	Always	Never	Never	Never	Sometimes
C24	Sometimes	Always	Sometimes	Always	Never	NA	Never	NA
C25	NA	Never	NA	Never	NA	Never	NA	Never
C26	Always	Always	Always	Always	NA	Sometimes	Always	Sometimes
C27	Almost always	Always	Almost always	Always	Never	Sometimes	Never	Never
C28	Always	Always	Always	Almost always	NA	Never	NA	Sometimes
C29	Always	Almost always	Always	Never	Sometimes	Never	Sometimes	Never

9e. 10-17 year-old children

Country	Weight		Length		Head circumference		MUAC	
	Ministry	IPA	Ministry	IPA	Ministry	IPA	Ministry	IPA
C1	NA	Always	NA	Always	NA	Sometimes	NA	Always
C2	Never	Always	Never	Always	Never	Never	Never	Sometimes
C3	Always	Always	Always	Always	Never	Sometimes	Never	Sometimes
C4	Always	Always	Always	Always	Never	Never	Don't know	Never
C5	NA	Always	NA	Always	NA	Sometimes	NA	Sometimes
C6	Always	Always	Always	Always	Never	Sometimes	Never	Never
C7	Always	Always	Always	Always	Never	Always	Sometimes	Never
C8	NA	Sometimes	NA	Sometimes	NA	Never	NA	Never
C9	Sometimes	Always	Never	Almost always	NA	Never	NA	Don't know
C10	Sometimes	Always	Sometimes	Almost always	Never	NA	Never	NA

	**Weight**		**Length**		**Head circumference**		**MUAC**	
**Country**	**Ministry**	**IPA**	**Ministry**	**IPA**	**Ministry**	**IPA**	**Ministry**	**IPA**
C11	NA	Never	NA	Never	NA	Never	NA	Never
C12	NA	Always	NA	Always	NA	Never	NA	Sometimes
C13	NA	NA	NA	NA	NA	NA	NA	NA
C14	Always	Always	Sometimes	Sometimes	Sometimes	Sometimes	Sometimes	Sometimes
C15	Always	Always	Always	Always	Never	NA	NA	NA
C16	Sometimes	Almost always	Sometimes	Sometimes	Never	Never	Never	Never
C17	Sometimes	Never	Sometimes	Never	NA	Never	NA	Never
C18	Don't know	Always	Don't know	Almost always	Don't know	Never	Don't know	Never
C19	Never	Always	Never	Always	Never	NA	Never	Always
C20	Always	Always	Always	Always	NA	Never	NA	Sometimes
C21	Sometimes	Sometimes	Sometimes	Sometimes	NA	Don't know	NA	Sometimes
C22	Sometimes	Always	Sometimes	Always	Never	NA	Never	Almost always
C23	Always	Always	Always	Always	Never	Never	Sometimes	Never
C24	Sometimes	Sometimes	Sometimes	Always	Never	NA	Never	NA
C25	NA	Never	NA	Never	NA	Never	NA	Never
C26	NA	Always	NA	Always	NA	Never	NA	Never
C27	Always	Always	Almost always	Always	Never	Never	Never	Never
C28	Always	Always	Always	Almost always	NA	Never	NA	Never
C29	Always	Almost always	Always	Never	Sometimes	Never	Sometimes	Never

12. What growth reference is used for comparison and calculating these anthropometric indices? (choose all that apply)

12a. under-five-year-old children

	World Health Organization		National reference		Other	
Country	Ministry	IPA	Ministry	IPA	Ministry	IPA
C1	X	X				
C2	X	X	X			
C3		X			X	
C4	X	X				X
C5	X	X				
C6		X			X	
C7	X	X	X	X		
C8	X	X	X			
C9	X	X	X			
C10	X	X				
C11	X	X				
C12	X	X				
C13	X			X		
C14	X	X				
C15	X	X	X		X	
C16	X	X		X		
C17	X	X				
C18	X	X				
C19	X	X				
C20	X	X				
C21	X	X				
C22	X	X		X		
C23	X	X	X	X		
C24	X	X				
C25	X	X		X		
C26	X	X				
C27	X	X				
C28	X	X				
C29	X	X	X	X		

12b. over-five-year-old children

Country	World Health Organization		National reference		Other	
	Ministry	IPA	Ministry	IPA	Ministry	IPA
C1	X	X				
C2		X				
C3	X			X		
C4	X	X				X
C5	X	X				
C6		X			X	
C7	X	X	X	X		
C8	X	X				
C9	X	X	X			
C10	X	X				
C11						X
C12		X				
C13						
C14			X	X		
C15	X	X	X		X	
C16		X		X		
C17	X	X				
C18	X	X				
C19	X	X				
C20	X	X				
C21	X	X				
C22	X	X		X		
C23	X	X	X	X		
C24	X	X				
C25						
C26		X				
C27	X	X				
C28	X	X				
C29	X			X		

15. What criteria are used to determine if there is a problem with the child’s growth*? (choose all that apply)
*A value that is below and above a nationally or internationally agreed screening cut-off. WAZ = weight-for-age Z-score, WLZ = weight-for-length Z-score, WHZ = weight-for-height Z-score, LAZ = length-for-age Z-score, HAZ = height-for-age Z-score, MUAC = Mid upper arm circumference

	**Attained WAZ**		**Attained WLZ/WHZ**		**Attained MUAC**		**Attained LAZ/HAZ**		**Parental -height adjusted LAZ/HAZ**		**Change in WHZ**		**Change in LAZ/HAZ**		**Other**	
**Country**	**Ministry**	**IPA**	**Ministry**	**IPA**	**Ministry**	**IPA**	**Ministry**	**IPA**	**Ministry**	**IPA**	**Ministry**	**IPA**	**Ministry**	**IPA**	**Ministry**	**IPA**
C1	X			X		X		X								
C2	X	X	X	X	X		X				X		X			
C3		X		X				X		X						
C4	X	X	X	X			X	X			X	X	X	X	X	X
C5	X	X	X	X	X	X	X	X								
C6	X		X													X
C7	X	X	X	X			X	X	X	X	X	X	X	X		
C8		X		X		X		X			X	X		X		
C9																
C10	X	X	X		X						X		X			
C11	X		X		X		X				X		X			X
C12	X	X	X	X	X	X	X	X				X				
C13	X	X	X		X	X	X									
C14		X		X		X		X		X		X		X	X	
C15		X		X		X		X		X		X		X	X	
C16	X		X	X	X	X	X									
C17	X	X	X		X		X									
C18			X	X		X		X		X						
C19	X	X		X	X	X		X								
C20	X	X	X	X		X	X	X	X		X	X	X			
C21	X	X	X		X		X									
C22	X	X	X				X									
C23	X	X	X	X	X	X	X	X	X	X	X	X	X	X		
C24					X						X					
C25	X	X	X	X	X	X	X									
C26	X		X	X	X	X										
C27	X	X	X	X	X	X	X	X		X	X	X	X	X		
C28	X	X					X	X					X			
C29	X	X	X				X	X		X	X	X	X	X		

16. What are the 1-3 most common actions in addition to counselling the parents, if a child is undergoing growth faltering*? Choose a maximum of 3 alternatives.
*Growth faltering means that the child is growing slower than expected. i.e.,S/he has slow weight gain or decreasing Z-score in weight-for-age (WAZ) or height for age (HAZ).

16a. 0-11 month-old children

	Nothing		Nutritional or other advice		Nutritional intervention		More frequent follow-up		Health intervention	
**Country**	**Ministry**	**IPA**	**Ministry**	**IPA**	**Ministry**	**IPA**	**Ministry**	**IPA**	**Ministry**	**IPA**
C1			X	X		X	X			
C2			X	X	X	X				X
C3				X	X		X	X	X	X
C4			X	X	X		X	X		X
C5			X	X		X	X			
C6			X			X		X	X	X
C7			X	X	X		X	X		
C8				X	X	X	X			
C9				X	X		X	X		X
C10			X		X	X		X		
C11			X	X					X	X
C12			X	X	X	X			X	
C13			X	X		X			X	
C14			X	X	X				X	X
C15					X		X	X		X
C16			X	X	X	X				X
C17			X	X	X	X	X	X		
C18			X	X			X	X	X	X
C19			X	X		X				X
C20			X	X		X	X		X	
C21				X	X	X		X	X	
C22			X	X	X	X				
C23			X	X		X	X	X		
C24			X	X	X			X	X	X
C25			X	X	X		X			
C26			X	X	X	X		X		
C27			X	X		X	X	X		
C28			X	X	X	X	X			
C29			X	X	X		X			X

16b. 12-23 month-old children

	Nothing		Nutritional or other advice		Nutritional intervention		More frequent follow-up		Health intervention	
**Country**	**Ministry**	**IPA**	**Ministry**	**IPA**	**Ministry**	**IPA**	**Ministry**	**IPA**	**Ministry**	**IPA**
C1			X	X		X	X			
C2			X	X	X	X				X
C3				X	X		X	X	X	X
C4			X	X	X		X	X		X
C5			X	X		X	X			
C6			X			X		X	X	X
C7			X	X	X		X	X		
C8				X	X	X	X			
C9			X	X	X		X	X		X
C10			X		X	X		X		
C11			X	X					X	X
C12			X	X	X	X			X	
C13			X	X		X			X	
C14			X	X	X				X	X
C15			X		X		X	X		X
C16			X	X	X	X				X

Country	Nothing		Nutritional or other advice		Nutritional intervention		More frequent follow-up		Health intervention	
	Ministry	IPA	Ministry	IPA	Ministry	IPA	Ministry	IPA	Ministry	IPA
C17			X		X	X	X			X
C18			X	X	X					X
C19			X	X	X	X				
C20			X	X		X	X		X	
C21				X	X	X			X	X
C22			X	X	X	X				
C23			X	X			X	X		X
C24			X	X	X			X	X	
C25			X		X	X	X			X
C26			X	X	X	X		X		
C27			X	X	X	X		X		
C28			X	X	X	X	X			
C29			X	X	X		X			X

16c. 2-4 year-old children

Country	Nothing		Nutritional or other advice		Nutritional intervention		More frequent follow-up		Health intervention	
	Ministry	IPA	Ministry	IPA	Ministry	IPA	Ministry	IPA	Ministry	IPA
C1				X		X				
C2			X	X	X	X				
C3				X	X		X		X	X
C4			X	X	X			X	X	X
C5			X	X		X	X			
C6						X		X	X	
C7			X	X	X		X	X		
C8				X	X		X			X
C9			X	X	X	X	X			X
C10			X	X	X	X		X		
C11			X	X					X	X
C12			X	X	X	X			X	
C13			X	X		X			X	
C14			X	X	X				X	X
C15			X		X		X	X		X
C16			X	X	X	X				X
C17			X		X	X	X			X
C18			X	X	X	X				X
C19			X	X	X	X				X
C20			X	X		X	X		X	
C21				X	X	X			X	
C22			X	X	X	X		X		
C23			X	X		X	X			X
C24			X	X	X			X	X	
C25										
C26			X	X	X	X		X		
C27			X	X	X	X		X		
C28			X	X	X	X	X			
C29			X	X	X		X			X

16d. 5-9 year-old children

Country	Nothing		Nutritional or other advice		Nutritional intervention		More frequent follow-up		Health intervention	
	Ministry	IPA	Ministry	IPA	Ministry	IPA	Ministry	IPA	Ministry	IPA
C1				X						X
C2			X	X	X	X				
C3				X	X		X		X	X
C4			X	X	X			X	X	X
C5				X		X				

	Nothing		Nutritional or other advice		Nutritional intervention		More frequent follow-up		Health intervention	
**Country**	**Ministry**	**IPA**	**Ministry**	**IPA**	**Ministry**	**IPA**	**Ministry**	**IPA**	**Ministry**	**IPA**
C6				X		X			X	
C7			X	X	X		X			X
C8				X	X		X			X
C9				X		X			X	X
C10			X	X		X				
C11										
C12				X		X				
C13				X						X
C14			X	X	X				X	X
C15			X	X			X	X	X	X
C16				X						X
C17			X		X		X			X
C18			X	X	X	X				X
C19	X			X		X				X
C20				X		X				X
C21			X	X					X	X
C22			X	X		X				
C23			X	X			X			X
C24			X		X	X		X	X	
C25										
C26			X	X	X	X		X		
C27			X	X			X	X	X	
C28			X	X		X			X	
C29			X	X			X		X	X

16e. 10-17 year-old children

	Nothing		Nutritional or other advice		Nutritional intervention		More frequent follow-up		Health intervention	
**Country**	**Ministry**	**IPA**	**Ministry**	**IPA**	**Ministry**	**IPA**	**Ministry**	**IPA**	**Ministry**	**IPA**
C1				X						X
C2			X	X	X	X				
C3				X	X				X	X
C4			X	X	X			X	X	X
C5				X		X				
C6				X					X	X
C7			X	X	X		X			
C8				X						X
C9				X		X				X
C10			X	X		X				
C11										
C12				X						X
C13										X
C14			X	X	X				X	X
C15			X	X			X	X	X	X
C16				X						X
C17			X		X		X			X
C18			X	X	X					X
C19	X			X						X
C20				X						X
C21			X	X	X				X	X
C22			X	X		X		X		
C23			X				X	X		X
C24			X	X	X			X	X	X
C25										
C26				X		X		X		
C27			X	X				X	X	X
C28			X	X		X			X	
C29			X	X			X		X	X

17. What are the 1-3 most common actions in addition to counselling the parents, if a child is identified as stunted*? Choose a maximum of 3 alternatives.
*Being stunted means that the child has low length-for-age Z score (LAZ).

17a. 0-11 month-old children

	Nothing		Nutritional or other advice		Nutritional intervention		More frequent follow-up		Health intervention	
**Country**	**Ministry**	**IPA**	**Ministry**	**IPA**	**Ministry**	**IPA**	**Ministry**	**IPA**	**Ministry**	**IPA**
C1			X	X		X	X			
C2			X	X	X	X				
C3				X		X	X		X	
C4				X	X		X	X	X	X
C5			X			X	X			X
C6								X	X	X
C7			X	X	X		X			X
C8				X	X	X	X		X	
C9			X	X	X	X	X	X		
C10			X	X	X	X				
C11		X	X							
C12			X	X	X	X			X	
C13			X	X		X				
C14			X	X	X				X	X
C15			X				X	X	X	X
C16			X	X	X					
C17			X			X		X	X	
C18			X	X		X	X	X		X
C19	X			X		X				
C20			X	X		X	X			X
C21			X		X	X		X		
C22			X	X		X	X	X		
C23			X	X		X				X
C24			X		X			X	X	X
C25			X	X	X	X	X			X
C26			X	X	X	X		X		
C27			X	X	X	X	X	X		
C28			X	X	X	X	X			
C29			X				X			X

17b. 12-23 month-old children

	Nothing		Nutritional or other advice		Nutritional intervention		More frequent follow-up		Health intervention	
**Country**	**Ministry**	**IPA**	**Ministry**	**IPA**	**Ministry**	**IPA**	**Ministry**	**IPA**	**Ministry**	**IPA**
C1			X	X		X	X			
C2			X	X	X	X				
C3				X		X	X	X	X	
C4					X		X	X	X	X
C5			X			X	X			X
C6				X		X			X	X
C7			X	X	X		X			X
C8				X	X	X			X	
C9			X	X	X	X	X	X		
C10			X		X	X		X		
C11		X	X							
C12			X	X	X	X			X	
C13			X	X		X				
C14			X	X	X				X	X
C15			X					X	X	X
C16			X	X	X					
C17			X		X	X				X

	Nothing		Nutritional or other advice		Nutritional intervention		More frequent follow-up		Health intervention	
**Country**	**Ministry**	**IPA**	**Ministry**	**IPA**	**Ministry**	**IPA**	**Ministry**	**IPA**	**Ministry**	**IPA**
C18			X	X	X	X		X	X	
C19	X			X		X				
C20			X			X	X		X	
C21			X	X	X	X		X		
C22			X	X		X	X	X		
C23			X	X		X			X	
C24			X		X			X	X	X
C25			X		X	X	X	X		X
C26			X	X	X	X		X		
C27			X	X	X	X	X			X
C28			X	X	X	X	X			X
C29			X				X	X	X	X

17c. 2-4 year-old children

	Nothing		Nutritional or other advice		Nutritional intervention		More frequent follow-up		Health intervention	
**Country**	**Ministry**	**IPA**	**Ministry**	**IPA**	**Ministry**	**IPA**	**Ministry**	**IPA**	**Ministry**	**IPA**
C1				X		X				
C2			X	X	X	X				
C3				X		X	X		X	X
C4					X		X	X	X	X
C5			X			X	X			X
C6				X		X		X	X	
C7			X	X	X		X			X
C8				X	X				X	X
C9			X	X	X	X	X			X
C10			X	X	X	X				
C11		X	X							
C12			X	X	X	X			X	
C13			X	X		X				
C14			X	X	X				X	X
C15			X					X	X	X
C16			X	X						
C17			X		X	X				X
C18			X	X	X			X	X	
C19	X			X		X				
C20			X	X			X		X	X
C21			X	X	X	X		X		
C22			X	X		X	X			
C23			X	X		X	X			
C24			X		X			X	X	X
C25										
C26			X	X	X	X		X		
C27			X	X	X	X	X	X		
C28			X	X	X	X	X			
C29			X	X			X		X	X

17d. 5-9 year-old children

	Nothing		Nutritional or other advice		Nutritional intervention		More frequent follow-up		Health intervention	
**Country**	**Ministry**	**IPA**	**Ministry**	**IPA**	**Ministry**	**IPA**	**Ministry**	**IPA**	**Ministry**	**IPA**
C1				X						X
C2			X	X	X	X				X
C3				X		X	X	X	X	
C4					X			X	X	X
C5						X				X
C6				X		X			X	

Country	Nothing		Nutritional or other advice		Nutritional intervention		More frequent follow-up		Health intervention	
	Ministry	IPA	Ministry	IPA	Ministry	IPA	Ministry	IPA	Ministry	IPA
C7			X	X	X		X			X
C8			X	X			X		X	X
C9				X		X		X	X	
C10	X			X		X				
C11		X								
C12				X		X				
C13				X						X
C14			X	X	X				X	X
C15			X	X					X	X
C16				X						
C17			X							X
C18			X	X	X			X	X	
C19	X			X		X				X
C20										X
C21			X							X
C22			X	X	X	X		X		
C23			X				X	X		X
C24			X		X	X		X	X	X
C25										
C26			X	X	X	X		X		
C27			X	X	X	X			X	X
C28			X	X		X		X	X	
C29			X	X			X		X	X

17e. 10-17 year-old children

Country	Nothing		Nutritional or other advice		Nutritional		More frequent follow-up		Health intervention	
	Ministry	IPA	Ministry	IPA	Ministry	IPA	Ministry	IPA	Ministry	IPA
C1				X						X
C2			X	X	X	X				X
C3				X		X		X	X	
C4					X			X	X	X
C5						X				X
C6				X		X			X	
C7			X	X	X		X			X
C8			X	X					X	X
C9				X		X				
C10	X			X		X				
C11		X								
C12				X						X
C13										X
C14			X	X	X				X	X
C15			X	X					X	X
C16				X						
C17	X									X
C18			X	X				X	X	
C19	X			X						X
C20										X
C21			X		X	X				X
C22			X	X	X	X		X		
C23			X				X	X		X
C24			X		X			X	X	X
C25										
C26				X		X		X		
C27				X	X	X	X		X	X
C28			X	X		X			X	X
C29			X	X			X		X	X

Appendix S3 in the **Online Supplementary Document.** International Growth Monitoring Survey Consortium members and their affiliations.

Osama Abdi, Ministry of Health, Somalia; Madina Ali Abdirahman, UNICEF, Somalia; Belen Aguirrezabalaga, Spanish Society of Primary Care Paediatrics, Spain; Basim Al-Zoubi, Ministry of Health, Jordan; Mona Alameh, Lebanese Paediatric Society, Lebanon; Cecilia D. Alinea, Philippine Paediatric Society, Philippines; Christine Jane B. Almira, National Nutrition Council, Philippines; Hind Alsharhan, Farwaniya Hospital and Ministry of Health, Kuwait; Abdulmajeed AlSubaihin, Department of Paediatrics, College of Medicine, King Saud University, Saudi Arabia; Rola Alzir, Emirates Paediatric Neonatal Medical Society, United Arab Emirates; Beatrice Amadi, Tropical Gastroenterology and Nutrition group, School of Medicine, University of Zambia and Zambia Paediatric Association, Zambia; Michael Anastasiades, Cyprus Paediatric Society, Cyprus; Kim Ang, National Paediatric Hospital, Cambodia; Ananias Antonio, Ministry of Health, Mozambique; Sofijanova Aspazija, Neonatal and Paediatric Intensive Care Unit, University Children's Hospital Skopje, North Macedonia; Simon Jonas Ategbo, Mother and Child University Hospital of the Jeanne Ebori Foundation, Gabon; Svitlana Austin, Paediatric Association of Zimbabwe, Zimbabwe; Damte Shimelis Awoke, Addis Ababa University, Ethiopia; Khamisa Ayoub, Ministry of Health, South Sudan; Ingrid Pamela Báez Echeverría, Ministry of Health, Ecuador; Shamsov Bakhtovar, Republican Scientific and Clinical Centre of Paediatrics and Child Surgery, Tajikistan; Cecilia Barragán, Pan American Health Organization/WHO, Ecuador; Nigar Bayramova, Azerbaijan Medical University and Azerbaijan Paediatric Society, Azerbaijan; Ramush Bejiqi, Paediatric Clinic, University Clinical Centre of Kosovo, Kosovo; Nellie VT Bell, University of Sierra Leone Teaching Hospitals Complex, Sierra Leone; Bruno Bindamba Senge, National Nutrition Program, Democratic Republic of the Congo; Nardos Birru, UNICEF, Ethiopia; Asma Bouaziz, Ministry of Health, Tunisia; María Catalina Carvajal, Technical Secretariat Ecuador Grows without Chronic Malnutrition, Ecuador; Alvin SM Chang, KK Women's and Children's Hospital, Singapore; Jean-Pierre Chanoine, University of British Columbia, Canada; Olga Cirstea, Moldavian Paediatric Society and Department of Paediatrics, Nicolae Testemitanu State University of Medicine, Republic of Moldova; Natália Maria Ferreira da Conceiçâo Rodrigues, Ministry of Health, Angola; Laura María Cristales Telón, Food Security and Nutrition Program, Guatemala; Lovely Daisy, Ministry of Health, Indonesia; Ibrahima Sory Diallo, Association Guinéenne de Pédiatrie, Guinea and Institute of Nutrition and Child Health, Guinea; Ana Lucía Díez Recinos, Paediatric Association of Guatemala, Guatemala; Željka Draušnik, Croatian Institute of Public Health, Croatia; Ekanem Ekure, Paediatric Association of Nigeria, Nigeria; Mercedes Esquivel Lauzurique, Cuban Society of Pediatrics, Cuba; Ali Faraj Ali Nassr, Libyan Paediatric Society, Libya; Julia Fernández Monge, Costa Rican Association of Paediatricians, Costa Rica; Kouéta Fla, Paediatric Society Of Burkina Faso, Burkina Faso; Amorissani Folquet, Ivoirian Paediatric association, Côte d'Ivoire; Christophe Gnimi, Ministry of Health, Congo; Jean Chrysostome Gody, Central African Paediatric Society, Central African Republic; Madonna Grimes, Ministry of Health, Guyana; Nadia Guellouz, Tunisian Society of Paediatrics, Tunisia; Sahar Idelbi, Syrian Paediatric

Association, Syria; Violeta Iotova, Medical University Varna, Bulgaria; Majed Abu Jaish, Paediatric Department, Al-Ittihad Hospital and Paediatric Society Palestine, Palestine; Tuomas Jartti, Department of Paediatrics and Adolescent Medicine, University of Turku and Turku University Hospital, Finland; Mari-Louie Jeffery, UNICEF, Namibia; Pawana Kayastha, Nepal Paediatric Society, Nepal; Mediatrice Kiburente, UNICEF, South Sudan; Georgios Konstantinidis, Pediatric Association of Serbia, Serbia; Anne Mei-Kwun Kwok, Hong Kong Children's Hospital, Hong Kong SAR, China; Maja Lang Morović, Croatian Institute of Public Health, Croatia; Agnès Linglart, University Paris Saclay, Le Kremlin-Bicêtre, France and AP-HP, Department of Endocrinology and Diabetology for Children, Bicêtre Paris-Saclay Hospital, France; Phim Loan, Ministry of Health, Cambodia; Kvashnina Lyudmila, Ukrainian Centre of Maternity and Childhood of the National Academy of Medical Sciences of Ukraine, Ukraine; Fiawoo Mawouto, Togolese Paediatric Society and Paediatric Department, Faculty of Health Sciences, University of Lomé, Togo; Emmie W Mbale, Paediatrics and Child Health Association, Malawi, Kamuzu University of Health Sciences and ALMA DELTAS, Malawi; Hubert Désiré Mbassi Awa, Cameroon Paediatric Association, Cameroon; María José Mendoza, UNICEF, Ecuador; Gladys Anabella Miranda Fuentes, Food Security and Nutrition Program, Guatemala; Ladda Mo-Suwan, Faculty of Medicine, Prince of Songkla University and Paediatric Society of Thailand, Thailand; Annang Giri Moelyo, Department of Child Health, Faculty of Medicine, Dr. Moewardi Hospital, Universitas Sebelas Maret, Indonesia; Claudia Montesinos Ramirez, Mexican Association of Paediatrics, Mexico; Paul Moscoso, Ecuadorian Society of Paediatrics, Ecuador; Bouraima Mouawiyatou, Ministry of Health, Togo; Yeva Movsesyan, Arabkir Medical Centre – Institute of Child and Adolescent Health and Armenian Paediatric Association, Armenia; Florence Mtawale, Ministry of Health, Zambia; Aida Mujkić, Croatian Paediatric Society, Croatia; Mehreen Mujtaba, Ministry of National Health Services, Regulation and Coordination, Pakistan; Aimée Mupuala, Society Paediatric Congo Democratique Republic, Democratic Republic of the Congo; Pius David Muzzazzi, Paediatric Association of Tanzania, Tanzania; Barbara Nalubanga, International Baby Food Action Network, Uganda; Leyla Namazova-Baranova, Union of Paediatricians of Russia, Russian Federation; Gorban Nataliya, Ukrainian Centre of Maternity and Childhood of the National Academy of Medical Sciences of Ukraine, Ukraine; Rute Neves, Portuguese Society of Paediatrics, Portugal; Pie Nibirantije, Burundian Association of Paediatrics, Burundi; Akhmedova Nilufar, Tashkent Paediatric Medical Institute, Uzbekistan; Fidele Nkezabahizi, Integrated National Programme for Food and Nutrition, Burundi; Dler Abdulkhaleq Nooruldeen, Kurdistan Paediatric Society, Iraq; Cecelia J. Nuta, Paediatrics Association of Liberia, Liberia; Azubuike Benjamin Nwako, Paediatric Association of Lesotho, Lesotho; Emmanuel Oppong, Paediatric Society of Ghana, Ghana; Fartun Abdullahi H Orey, Department of Paediatrics and Child Health, Dr Sumait Hospital, Faculty of Medicine and Health Sciences, SIMAD University, Somalia; Altagracia Páez, Dominican Society of Paediatrics, Dominican Republic; Huynh Nam Phuong, National Institute of Nutrition, Vietnam; Mariana del Pino, Argentina Society of Paediatrics, Argentina; Doina-Anca Plesca, Romanian Paediatric Society, Romania; Jorge Rada Noriega, UNICEF, Plurinational State of Bolivia; Tahiana Razafindrakoto, Ministry of Public Health, Madagascar; Macarena

Riquelme Rivera, Undersecretary of Healthcare Networks, Chile; María Inés Romero, Ministry of Health, Chile; Marysol Ruilova, UNICEF, Ecuador; Elieth Rumanyika, Ministry of Health, Tanzania; Masood Sadiq, Pakistan Paediatric Association and University of Child Health Sciences, Children’s Hospital, Pakistan; Haroon Saloojee, University of the Witwatersrand, South Africa; Setshedi Sebata, Botswana Paediatric Association, Botswana; Amela Selimovic, Clinic for Children’s Diseases, University Clinical Centre Tuzla, Bosnia and Herzegovina; Virendra RS Singh, Paediatric Society of Trinidad and Tobago, Trinidad and Tobago; Selva Kumar Sivapunniam, Malaysian Paediatric Association, Malaysia; Santosh Soans, Department of Paediatrics, A.J. Institute of Medical Sciences & Research Centre, India; Dirceu Sole, Brazilian Society of Paediatrics, Brazil and Paulista School of Medicine, Federal University of São Paulo, Brazil; Félix Sonon, National Agency for Food and Nutrition, Benin; Chan Sophal, Ministry of Health, Cambodia; Adriana Sosa Botana, Ministry of Public Health, Uruguay; Shayirbek Alibaevich Sulaimanov, Union of Children’s Doctors of the Kyrgyz Republic, Kyrgyzstan; Mariam Sylla, Association Malienne de Pédiatrie, Mali; Lila Bikram Thapa, Ministry of Health and Population, Nepal; Fathimath Thohira, Health Protection Agency, Maldives; Vaidotas Urbonas, Clinic of Children Diseases, Medical Faculty, Vilnius University, Lithuania; Ruth Aburto Vallejos, Undersecretariat of Public Health, Chile; Laura Elizabeth Vásquez Muñoz, Ministry of Public Health, Ecuador; Verónica Véliz Rojas, Undersecretary of Healthcare Networks, Chile; Sergio Venturino, Uruguayan Society of Paediatrics, Uruguay; V Pujitha Wickramasinghe, Department of Paediatrics, Faculty of Medicine, University of Colombo, Sri Lanka; Wei Xiang, Child Health Care Group, Paediatrician Branch, Chinese Medical Association, China; Hamda Omar Yousuf, UNICEF, Somalia; Antipkin Yuriy, Ukrainian Centre of Maternity and Childhood of the National Academy of Medical Sciences of Ukraine, Ukraine; Fatima Mohammed Yusuf, Nutrition Services Division, Nigeria; Leela Keculah Zaizay, UNICEF, Papua New Guinea; Yue Zhang, National Centre for Women and Children’s Health, China; Magaly Zurita Villazón, Bolivian Paediatric Society, Plurinational State of Bolivia; Siniketiwe Zwane, Eswatini National Nutrition Council, Eswatini.
